# Comparative Effectiveness of Adalimumab vs Tofacitinib in Patients With Rheumatoid Arthritis in Australia

**DOI:** 10.1001/jamanetworkopen.2023.20851

**Published:** 2023-06-29

**Authors:** Claire T. Deakin, Bianca L. De Stavola, Geoffrey Littlejohn, Hedley Griffiths, Sabina Ciciriello, Peter Youssef, David Mathers, Paul Bird, Tegan Smith, Catherine O’Sullivan, Tim Freeman, Dana Segelov, David Hoffman, Shaun R. Seaman

**Affiliations:** 1 OPAL Rheumatology Ltd, Sydney, New South Wales, Australia; 2 Centre for Adolescent Rheumatology Versus Arthritis at University College London, University College London Hospitals, Great Ormond Street Hospital and University College London, London, United Kingdom; 3 National Institute of Health Research Biomedical Centre at Great Ormond Street Hospital, London, United Kingdom; 4 Population, Policy and Practice Research and Teaching Department, UCL Great Ormond Street Institute of Child Health, London, United Kingdom; 5 Department of Medicine, Monash University, Clayton, Victoria, Australia; 6 Barwon Rheumatology Service, Geelong, Victoria, Australia; 7 Royal Melbourne Hospital, Melbourne, Victoria, Australia; 8 Royal Prince Alfred Hospital, Sydney, New South Wales, Australia; 9 University of Sydney, Sydney, New South Wales, Australia; 10 Georgetown Arthritis, Newcastle, New South Wales, Australia; 11 University of New South Wales, Kensington, New South Wales, Australia; 12 Software for Specialists Pty Ltd, Sydney, New South Wales, Australia; 13 MRC Biostatistics Unit, University of Cambridge, Cambridge, United Kingdom

## Abstract

**Importance:**

There is a need for observational studies to supplement evidence from clinical trials, and the target trial emulation (TTE) framework can help avoid biases that can be introduced when treatments are compared crudely using observational data by applying design principles for randomized clinical trials. Adalimumab (ADA) and tofacitinib (TOF) were shown to be equivalent in patients with rheumatoid arthritis (RA) in a randomized clinical trial, but to our knowledge, these drugs have not been compared head-to-head using routinely collected clinical data and the TTE framework.

**Objective:**

To emulate a randomized clinical trial comparing ADA vs TOF in patients with RA who were new users of a biologic or targeted synthetic disease-modifying antirheumatic drug (b/tsDMARD).

**Design, Setting, and Participants:**

This comparative effectiveness study emulating a randomized clinical trial of ADA vs TOF included Australian adults aged 18 years or older with RA in the Optimising Patient Outcomes in Australian Rheumatology (OPAL) data set. Patients were included if they initiated ADA or TOF between October 1, 2015, and April 1, 2021; were new b/tsDMARD users; and had at least 1 component of the disease activity score in 28 joints using C-reactive protein (DAS28-CRP) recorded at baseline or during follow-up.

**Intervention:**

Treatment with either ADA (40 mg every 14 days) or TOF (10 mg daily).

**Main Outcomes and Measures:**

The main outcome was the estimated average treatment effect, defined as the difference in mean DAS28-CRP among patients receiving TOF compared with those receiving ADA at 3 and 9 months after initiating treatment. Missing DAS28-CRP data were multiply imputed. Stable balancing weights were used to account for nonrandomized treatment assignment.

**Results:**

A total of 842 patients were identified, including 569 treated with ADA (387 [68.0%] female; median age, 56 years [IQR, 47-66 years]) and 273 treated with TOF (201 [73.6%] female; median age, 59 years [IQR, 51-68 years]). After applying stable balancing weights, mean DAS28-CRP in the ADA group was 5.3 (95% CI, 5.2-5.4) at baseline, 2.6 (95% CI, 2.5-2.7) at 3 months, and 2.3 (95% CI, 2.2-2.4) at 9 months; in the TOF group, it was 5.3 (95% CI, 5.2-5.4) at baseline, 2.4 (95% CI, 2.2-2.5) at 3 months, and 2.3 (95% CI, 2.1-2.4) at 9 months. The estimated average treatment effect was −0.2 (95% CI, −0.4 to −0.03; *P* = .02) at 3 months and −0.03 (95% CI, −0.2 to 0.1; *P* = .60) at 9 months.

**Conclusions and Relevance:**

In this study, there was a modest but statistically significant reduction in DAS28-CRP at 3 months for patients receiving TOF compared with those receiving ADA and no difference between treatment groups at 9 months. Three months of treatment with either drug led to clinically relevant average reductions in mean DAS28-CRP, consistent with remission.

## Key Points

**Question:**

What is the effectiveness of adalimumab (ADA) compared with tofacitinib (TOF) for treatment of rheumatoid arthritis in routine clinical practice?

**Findings:**

In this comparative effectiveness study of 842 patients with rheumatoid arthritis in Australia, TOF was favored slightly at 3 months vs ADA, but there was no difference in scores between patients receiving TOF and those receiving ADA at 9 months.

**Meaning:**

This study showed similar treatment effects for TOF and ADA, which is consistent with data from a randomized trial and current European Alliance of Associations for Rheumatology treatment guidelines.

## Teaser

This comparative effectiveness reseach study assesses the effecetiveness of adalimumab vs tofacitinib for treatment of rheumatoid arthiritis in the clinical setting.

## Introduction

In the past 20 years, the availability of tumor necrosis factor inhibitors (TNFis) and other biologic disease-modifying antirheumatic drugs (bDMARDs) has transformed treatment for patients with rheumatoid arthritis (RA). More recently, targeted synthetic DMARDs (tsDMARDs), including janus kinase inhibitors (JAKis), have become available and are considered equivalent to bDMARDs for patients with moderate to severe disease refractory to conventional synthetic DMARD (csDMARD) therapy.^1^

Although trials directly comparing specific b/tsDMARDs head-to-head are limited, a double-blind phase 3b/4 randomized clinical trial^2^ showed that tofacitinib (TOF) combined with methotrexate was noninferior to adalimumab (ADA) combined with methotrexate. Other trials have demonstrated the efficacy of JAKi therapy in patients who have not responded to methotrexate or TNFi therapy.^3,4^ Drug retention was longer for TOF compared with TNFis in an observational study of 4023 treatment courses that combined multiple lines of therapy.^5^ Similar outcomes have been described for TOF and JAKi therapy compared with TNFi therapy.^6,7^ To our knowledge, there are no observational studies evaluating the effectiveness of a JAKi drug compared with a TNFi drug in a b/tsDMARD-naive patient population.

Registries and real-world data sets (RWDs), which include routinely collected data such as electronic medical records (EMRs) and medical claims data, are a valuable source of information for understanding the effectiveness of treatments. There is increasing recognition of the complementary role for real-world evidence based on analyses of RWDs in health care and regulatory decision-making.^8^ However, there are significant challenges to the reliability of comparative effectiveness studies using RWDs.^9^ In this study, RWD refers to clinical records in patients’ EMRs that are routinely collected at the point of care. Outcomes are often recorded incompletely in registries and EMRs, patients are not randomized to treatment groups, and differences between groups need to be accounted for. There can also be differential durations of follow-up and attrition.

Target trial emulation (TTE) is a framework for comparative effectiveness analyses whereby principles from the design of randomized clinical trials (RCTs) are applied to observational research by making explicit the design of the trial that is intended to be emulated.^10^ In this study, we sought to emulate an RCT of ADA vs TOF in b/tsDMARD-naive patients with RA in the Optimising Patient Outcomes in Australian Rheumatology (OPAL) data set using an intention-to-treat analysis.^11^ To generate evidence from a large observational RWD, we developed a methodological approach to address the challenges of missing baseline and outcome data and nonrandomized treatment assignment. Our approach aimed to avoid the selection bias that could result from excluding patients with missing outcomes.

## Methods

Detailed technical methods of this comparative effectiveness study are described in eMethods 1 through 7 and the eAppendix in Supplement 1. Ethics approval was obtained for research using deidentified data in the OPAL data set from the University of New South Wales Human Research Ethics Committee (HC17799) and for the specific protocol (HC210647). Patients consented to their deidentified EMR data being used for research via an opt-out consent model. This report followed the International Society for Pharmacoeconomics and Outcomes Research (ISPOR) reporting guideline.

### Design

The prespecified protocol for the target trial to be emulated is described in eMethods 1 in Supplement 1. In brief, the analysis was designed to emulate an RCT of ADA vs TOF in patients with RA who were new users of a b/tsDMARD using an intention-to-treat analysis. Mean disease activity score in 28 joints using C-reactive protein (DAS28-CRP) was assessed at 3 and 9 months after treatment with ADA or TOF was initiated.

### Participants

Eligible patients in the emulated target trial were adults aged 18 years or older who were diagnosed with RA; whose first visit occurred between April 1, 2015, and January 1, 2021; who had no prior recorded b/tsDMARD; who had at least 6 months from their first recorded visit until baseline; and who had at least 6 months of treatment with a csDMARD immediately prior to baseline. These criteria defined (or enriched for) a cohort of new users who were b/tsDMARD naive based on their EMR and the government criteria for b/tsDMARD reimbursement. Patients were excluded if they did not have at least 1 component of the DAS28-CRP recorded at baseline, 3 months, or 9 months.

### Interventions

Patients initiated treatment with either ADA (40 mg every 14 days) or TOF (10 mg daily) and, in the target trial, would be expected to continue treatment during follow-up unless an adverse event or contraindication occurred. The limited duration of availability of the biosimilar for ADA meant that all ADA interventions were the originator and not the biosimilar.

### End Point

The primary outcome was disease activity at 3 and 9 months after initiating treatment. The average treatment effect (ATE) was defined as the difference in mean DAS28-CRP among patients receiving TOF compared with those receiving ADA at 3 and 9 months.

These time points were selected because joint counts and pathologic markers are assessed for government reimbursement at 3 and 9 months. American College of Rheumatology response of at least 50% was used in the trial of ADA and TOF.^2^ However, its calculation requires multiple variables that are missing in the OPAL data set. Therefore, DAS28-CRP, used in other landmark trials for RA,^12,13^ was selected as a composite outcome that represents clinician- and patient-assessed disease and an objective pathologic marker.

### Setting and Data Source

This analysis used the multicenter OPAL data set, which includes the EMRs for 216 138 patients with rheumatic conditions treated by 112 rheumatologists across Australia at 43 different clinics since 2004. In Australia, government reimbursement is available for b/tsDMARDs for patients with moderate to severe disease who have not responded to at least 6 months of treatment with csDMARDs, and physicians can prescribe the b/tsDMARD that fits the patient’s clinical need. As such, there are no binding guidelines as to the order in which a b/tsDMARD class can be prescribed. Response to treatment is assessed at 3 months and at 6-month intervals thereafter. Tofacitinib is approved by the Australian Therapeutic Goods Administration for use for RA at a dosage of 5 mg twice daily.

The deidentified data in the OPAL data set include demographics, disease history, disease activity measures, comorbidities, pathology, medications, patient-reported outcomes, and characteristics of the treating rheumatologist. Data were collected from April 1, 2015, to June 30, 2021. A more detailed description of the data set and a summary of the characteristics of the variables used in this analysis are given in eMethods 2 in Supplement 1.

### Safety

Treatment cessations due to an adverse reaction were described for all patients who satisfied the inclusion criteria. Treating physicians have discretion to record an adverse reaction to a medication in the EMR, and there may have been unrecorded adverse reactions. All recorded adverse reactions that were considered more serious than nonserious were described. An adverse reaction recorded using the *International Statistical Classification of Diseases and Related Health Problems, Tenth Revision (ICD-10)* codes C* or D00-D48 was considered to be cancer.^14^ A major cardiovascular event (MACE) was defined according to previously published *ICD-10* codes for MACE in the context of administrative data sets and rheumatology: I21-I24 (myocardial infarction); I63-I66 (stroke); I11, I50, and I97.1 (heart failure); and Z95 (coronary artery bypass grafting).^15,16^

### Patient Involvement

Patients were not involved in the design of this study or consulted during selection of outcomes or the interpretation of findings. Patients will be involved in the dissemination of this research.

### Statistical Analysis

**Figure 1.**
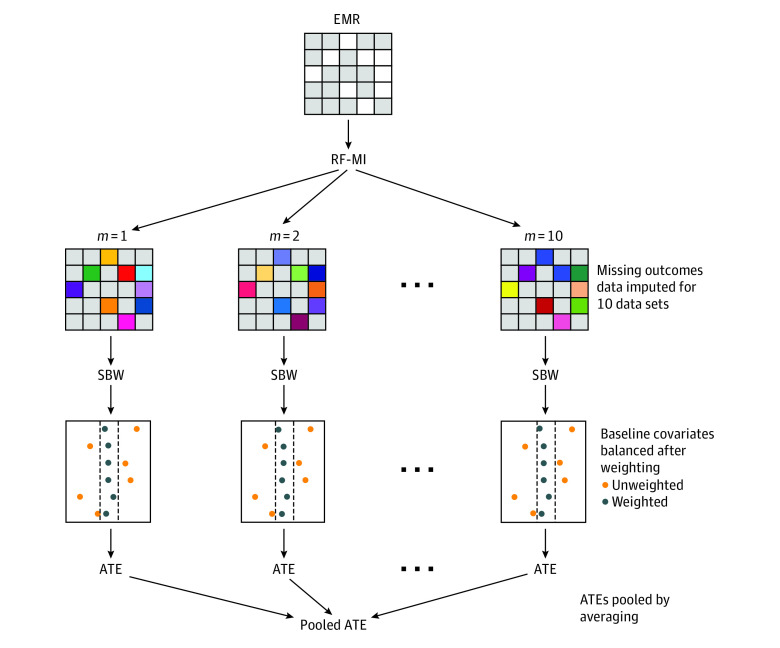
Multistep Analytical Procedure Developed to Estimate the Average Treatment Effect (ATE) of Tofacitinib (TOF) Compared With Adalimumab (ADA) Random forest multiple imputation (RF-MI) was used to impute plausible values for missing data in the original electronic medical record (EMR) data set. Stable balancing weights (SBWs) were used to balance the baseline characteristics of the ADA and TOF treatment groups. Gray squares in the grid that represents EMR indicate complete data items and white squares, missing data items. In the complete data sets generated by RF-MI, colored squares represent the imputed values. The different colors used for the same data item in different data sets indicate that imputed values were slightly different in each data set. The covariate balance plots indicate that after SBWs were applied, the mean standardized difference in baseline characteristics between the treatment groups was close to 0 compared with before weighting. The ATE was then calculated separately in each imputed data set before pooling to generate a final estimate.

A multistep method was developed to address the challenges of missing disease activity data and nonrandomized treatment assignment. Analysis that only uses complete cases could lead to selection bias, and thus, multiple imputation was used instead of complete-case analysis. An overview of the methods is presented in Figure 1, and the details are fully described in eMethods 1 through 7 and the eAppendix in Supplement 1.

In brief, multiple imputation by chained equations using the random forest algorithm under the missing-at-random assumption was used to impute missing data for the components of the DAS28-CRP at baseline and follow-up, generating 10 imputed data sets.^17^ Patients with no observed DAS28-CRP components at follow-up were then excluded after multiple imputation because these patients would not be informative about the treatment effect. Stable balancing weights (SBWs) were then used to account for differences in baseline characteristics between the included patients receiving ADA and the total eligible patients and between the included patients receiving TOF and the total eligible patients.^18^ Balance for the baseline characteristics was evaluated, including calculating standardized mean differences between the treatment groups, which express these differences in terms of the observed SDs in the sample of eligible patients. The difference in weighted DAS28-CRP was then calculated at 3 and 9 months within each imputed data set, and these estimates were pooled using Rubin rules to yield single point estimates at 3 and 9 months.^19^ The whole procedure was then bootstrapped using 1000 bootstrap samples to generate a 95% CI for the estimates using the percentile method.^20^

All analyses were performed using R, version 4.0.2 (R Project for Statistical Computing). Differences in mean DAS28-CRP at follow-up were assessed using a 2-sided test and a significance level of *P* < .05. eMethods 1 through 7, eResults 1 through 4, and the eAppendix in Supplement 1 give further details of the analysis, including the R code and checks of the performance of the multiple imputation algorithm and the balance between the treatment groups.

## Results

### Patients

**Figure 2.**
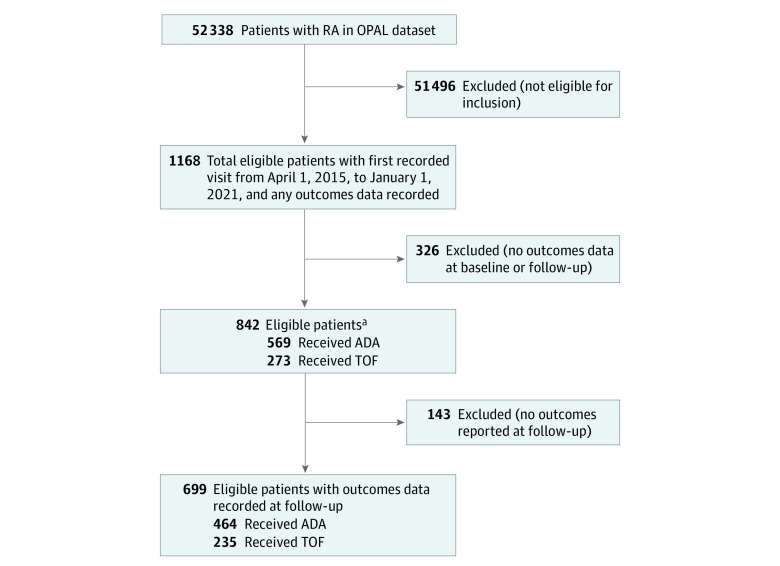
Flow Diagram of Inclusion and Exclusion of Patients with Rheumatoid Arthritis (RA) in the Optimising Patient Outcomes in Australian Rheumatology Data Set Stable balancing weights were used to make each treatment group balanced with each other and the 842 total eligible patients. ADA indicates adalimumab; TOF, tofacitinib. ^a^Multiple imputation was applied to these patients.

Of the 52 338 patients with RA in the OPAL data set, 842 eligible b/tsDMARD-naive patients who were new starters of ADA (n = 569; 387 [68.0%] female; 175 [30.8%] male; median age, 56 years [IQR, 47-66 years]) or TOF (n = 273; 201 [73.6%] female; 72 [26.4%] male; median age, 59 years [IQR, 51-68 years]) were identified (Figure 2). Patients without any components of DAS28-CRP recorded at baseline, 3 months, or 9 months who were otherwise eligible new starters were excluded (n = 339). The baseline characteristics of the excluded patients are described in eMethods 2 in Supplement 1.

### Baseline Data

Before SBWs were applied, there were small differences between the treatment groups (Table). For example, patients in the TOF group were slightly older and more likely to be female, to be located in states other than Victoria, to be located in outer regional or remote areas, and to have prior kidney disease. The TOF group also included a lower percentage of patients treated at a clinic with a nurse and a higher percentage of patients treated by rheumatologists with more years of experience, a higher overall tendency to prescribe b/tsDMARDs in their practice, and a lower overall tendency to complete the patient global score. There were small differences between the treatment groups in the csDMARD drugs that had been prescribed to patients prior to baseline.

**Table.** Baseline Characteristics of Patients With RA Treated With ADA or TOF

Characteristic	Patients with RA^a^	
	Received ADA (n = 569)	Received TOF (n = 273)
Age, median (IQR) [range]	56 (47-66) [18-88]	59 (51-68) [21-86]
Gender, No./total No. (%)^b^		
Female	387/562 (68.0)	201/273 (73.6)
Male	175/562 (30.8)	72/273 (26.4)
State, No./total No. (%)^c^		
Victoria	288/568 (50.6)	73/272 (26.7)
New South Wales	149/568 (26.2)	108/272 (39.6)
Australian Capital Territory	42/568 (7.4)	25/272 (9.2)
Tasmania	20/568 (3.5)	22/272 (8.1)
Queensland and Western Australia	69/568 (12.1)	44/272 (16.1)
Regional location, No./total No. (%)^d^		
Major cities	355/568 (62.4)	165/273 (60.4)
Inner regional	166/568 (29.2)	68/273 (24.9)
Outer regional and remote	47/568 (8.3)	40/273 (14.7)
Disease duration recorded in EMR, median (IQR) [range], y	1.0 (0.7-1.7) [0.5-5.3]	1.2 (0.8-2) [0.5-5.6]
Year of treatment start		
2015 and 2016	64 (11.2)	29 (10.6)
2017	110 (19.3)	60 (22.0)
2018	104 (18.3)	73 (26.7)
2019	118 (20.7)	65 (23.8)
2020	141 (24.8)	39 (14.3)
2021	32 (5.6)	7 (2.6)
DAS28-CRP components, median (IQR) [range]		
SJC28^e^	13 (6-22) [0-28]	12 (5-22) [0-28]
TJC28^e^	14 (7-22) [0-28]	12 (6-22) [0-28]
Patient global score^f^	60 (46.5-75) [0-100]	51 (30.5-74.2) [0-100]
CRP level^g^	6 (3-14) [1-221]	6 (3-14.7) [1.5-169]
Physician global score, median (IQR) [range]^h^	50 (40-70) [0-100]	50 (30-70) [3-100]
ESR, median (IQR) [range]^i^	10 (5-22.2) [1-106]	11 (5-28) [1-107]
DAS28-CRP, median (IQR) [range]^i^	5.7 (4.7-6.4) [1.2-7.8]	5.4 (4.2-6.4) [1.4-8.1]
Nurse at clinic		
Yes	284 (49.9)	74 (27.1)
No	285 (50.1)	199 (72.9)
Practitioner gender		
Female	230 (40.4)	117 (42.9)
Male	339 (59.6)	156 (57.1)
Practitioner experience, y		
0-15	187 (32.9)	74 (27.1)
16-30	226 (39.7)	142 (52)
>30	156 (27.4)	57 (20.9)
Practitioner overall tendency to complete patient global score, %		
0-75	219 (38.5)	126 (46.2)
76-100	350 (61.5)	147 (53.8)
Practitioner overall tendency to prescribe b/tsDMARDs, % total prescriptions		
0-25	494 (86.8)	216 (79.1)
26-100	75 (13.2)	57 (20.9)
Prior kidney disease		
Yes	229 (40.2)	130 (47.6)
No	340 (59.8)	143 (52.4)
Prior treatment		
Methotrexate		
Yes	355 (62.4)	184 (67.4)
No	214 (37.6)	89 (32.6)
Hydroxychloroquine		
Yes	204 (35.9)	130 (47.6)
No	365 (64.1)	143 (52.4)
Leflunomide		
Yes	161 (28.3)	95 (34.8)
No	408 (71.7)	178 (65.2)
Sulfasalazine		
Yes	158 (27.8)	74 (27.1)
No	411 (72.2)	199 (72.9)
Oral corticosteroids		
Yes	324 (56.9)	159 (58.2)
No	245 (43.1)	114 (41.8)
Concomitant treatment		
Methotrexate		
Yes	151 (26.5)	79 (28.9)
No	418 (73.5)	194 (71.1)
Hydroxychloroquine		
Yes	133 (23.4)	69 (25.3)
No	436 (76.6)	204 (74.7)
Leflunomide		
Yes	101 (17.8)	49 (17.9)
No	468 (82.2)	224 (82.1)
Sulfasalazine		
Yes	89 (15.6)	39 (14.3)
No	480 (84.4)	234 (85.7)
Oral corticosteroids		
Yes	282 (49.6)	131 (48.0)
No	287 (50.4)	142 (52.0)

Abbreviations: ADA, adalimumab; b/tsDMARDs, biologic or targeted synthetic disease-modifying antirheumatic drugs; CRP, C-reactive protein; DAS28-CRP, disease activity score in 28 joints using C-reactive protein; EMR, electronic medical record; ESR, erythrocyte sedimentation rate; RA, rheumatoid arthritis; SJC28, swollen joint count based on 28-joint assessment; TJC28, tender joint count based on 28-joint assessment; TOF, tofacitinib.

^a^ Data are presented as number (percentage) of patients unless otherwise indicated.

^b^ Data were missing for 7 patients in the ADA group (1.2%) and 0 patients in the TOF group.

^c^ Data were missing for 1 patient in the ADA group (0.2%) and 1 patient in the TOF group (0.4%).

^d^ Data were missing for 1 patient in the ADA group (0.2%) and 0 patients in the TOF group.

^e^ Data were missing for 68 patients in the ADA group (12.0%) and 40 in the TOF group (14.7%).

^f^ Data were missing for 213 patients in the ADA group (37.4%) and 115 in the TOF group (42.1%).

^g^ Data were missing for 75 patients in the ADA group (13.2%) and 45 in the TOF group (16.5%).

^h^ Data were missing for 210 patients in the ADA group (36.9%) and 118 in the TOF group (43.2%).

^i^ Data were missing for 217 patients in the ADA group (38.1%) and 120 in the TOF group (44.0%).

**Figure 3.**
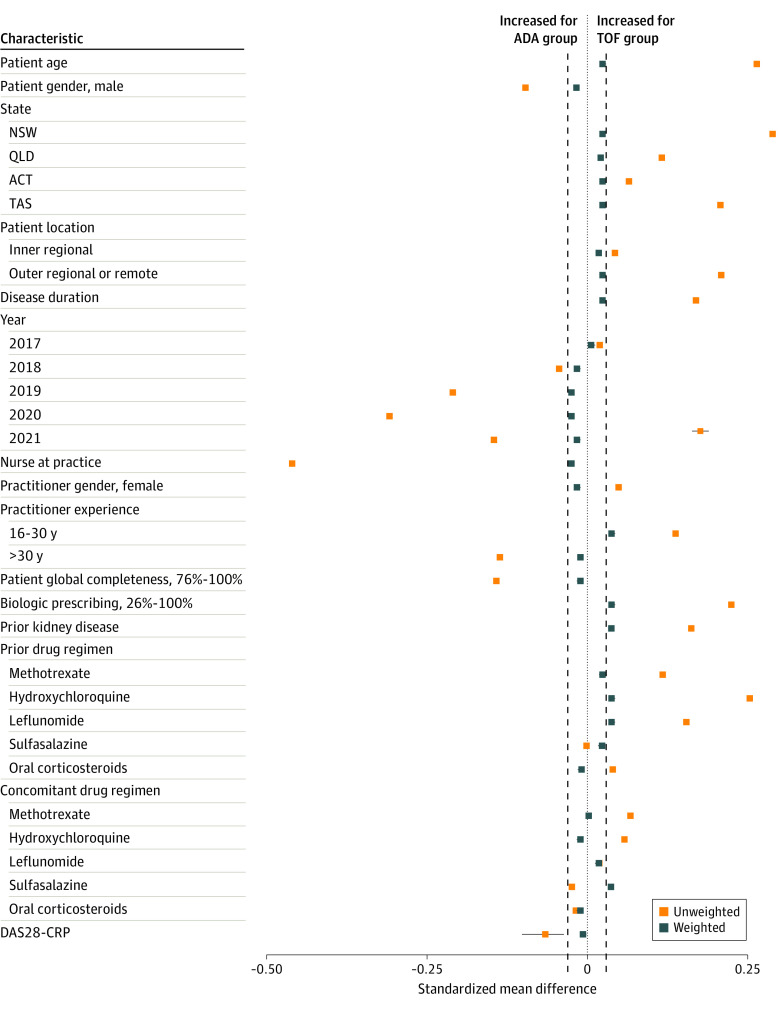
Standardized Mean Difference in Baseline Characteristics of Adalimumab (ADA) and Tofacitinib (TOF) Treatment Groups Before and After Weighting Error bars for disease activity score in 28 joints using C-reactive protein (DAS28-CRP) represent the minimum and maximum standardized mean difference values across 10 imputed data sets. The standardized mean difference is the difference between treatment groups in the mean for each covariate divided by its SD for the entire sample. The vertical dashed black lines indicate standardized mean differences of −0.03 and 0.03. ACT indicates Australian Capital Territory; NSW, New South Wales; QLD, Queensland and Western Australia; and TAS, Tasmania.

After SBWs were applied, the standardized mean differences in baseline characteristics were between −0.03 and 0.03, within the conventional threshold of 0.1 for propensity score matching (Figure 3). This indicated that the treatment groups were balanced on these measured characteristics using the SBW method.

### Estimated Comparative Effectiveness

After weighting, mean DAS28-CRP decreased from 5.3 (95% CI, 5.2-5.4) at baseline to 2.6 (95% CI, 2.5-2.7) at 3 months and 2.3 (95% CI, 2.2-2.4) at 9 months in the ADA group (eResults 5 in Supplement 1). Mean DAS28-CRP decreased from 5.3 (95% CI, 5.2-5.4) at baseline to 2.4 (95% CI, 2.2-2.5) at 3 months and 2.3 (95% CI, 2.1-2.4) at 9 months in the TOF group. These follow-up values are consistent with the threshold for remission (≤2.6).^21^

**Figure 4.**
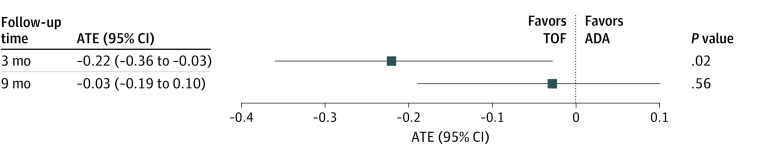
Estimated Average Treatment Effect (ATE) for Tofacitinib (TOF) Compared With Adalimumab (ADA) at 3 and 9 Months Squares represent ATEs, with horizontal lines representing 95% CIs based on bootstrap distributions of the estimates.

The ATE for TOF compared with ADA was −0.2 (95% CI, −0.4 to −0.03; *P* = .02) at 3 months and −0.03 (95% CI, −0.2 to 0.1; *P* = .60) at 9 months (Figure 4). This indicated that patients who received TOF had, on average, slightly lower DAS28-CRP at 3 months compared with patients who received ADA, but there was no difference in DAS28-CRP between the treatment groups at 9 months.

### Safety

Due to recent concerns about MACE and cancer associated with JAKi drugs,^22^ any cessations of ADA or TOF due to an adverse event are described in eResults 6 in Supplement 1 for all eligible patients (n = 842) and patients who had been excluded due to missing data on the components of the DAS28-CRP (n = 326). For these 1168 patients, there were 28 recorded treatment cessations due to adverse reactions in the ADA group (3.8%) and 19 in the TOF group (4.4%). Median follow-up in the ADA group was 2.1 years (IQR, 0.9-3.4 years) and in the TOF group was 2.5 years (IQR, 1.4-3.4 years).

There were no adverse reactions recorded using *ICD-10* codes for cancer and 1 (0.2%) for MACE (a medically significant case of embolic stroke in the TOF group). Additionally, there was 1 case of pulmonary embolism in each of the ADA (0.1%) and TOF (0.2%) treatment groups, the former of which was life-threatening but considered unrelated to treatment. There was 1 case of deep vein thrombosis in the TOF group.

## Discussion

In this comparative effectiveness study using the TTE framework, we found a modest but statistically significant reduction in disease activity associated with TOF compared with ADA at 3 months and no difference between drugs at 9 months in patients with RA who were b/tsDMARD naive. These results may be generalizable to patients with RA who have not responded to csDMARD therapy and are eligible to initiate their first b/tsDMARD, subject to the limitations of the study. Although the outcomes, time points, and superiority design differ, the small effect size in our analysis is consistent with the previous findings of noninferiority in the American College of Rheumatology response of at least 50% at 6 months for these drugs in a clinical trial.^2^ Our findings are also consistent with an observational study that combined multiple lines of therapy and showed slightly longer drug retention for TOF therapy compared with TNFi therapy.^5^ Our study benefitted from the large number of patients with RA in the OPAL data set, which enabled the analysis to focus on the effectiveness of TOF vs ADA received as the first b/tsDMARD. Our findings support the 2019 European Alliance of Associations for Rheumatology (EULAR) recommendations, which treat JAKis and bDMARDs as equivalent when used as first-line therapy.^1^

Observational studies are an important supplement to RCTs for assessing whether trial findings can be reproduced in everyday practice in a less restricted patient population and for guiding treatment decisions that occur outside the idealized setting of a trial.^23^ Given the well-known limitations of observational research, the need for quality in comparative effectiveness studies has been recognized.^9^ Although there have been few applications of the TTE framework in rheumatology,^24,25,26^ there have been numerous studies using propensity score methods, including propensity score matching and inverse probability of treatment weighting,^6,27,28,29,30,31,32,33,34^ and some of these are limited by time-related biases, such as comparing drugs or classes received as different lines of therapy.^35^ Covariate balancing is an alternative approach to propensity score methods and can lead to better balance while avoiding some of the known pitfalls of propensity score methods, such as model misspecification and dispersed weights.^36,37^ Additionally, this study dealt with the challenge of missing outcomes data, which is a source of bias not often addressed in observational studies.

Safety signals, although typically rare, are important in connection with ADA and TOF, especially in light of the relatively higher risks of MACE and cancer associated with JAKi therapy compared with TNFi therapy.^22^ Although the rates of MACE and other cardiovascular events in our study are consistent with those previously reported, this study was not set up to address safety, and we only described recorded adverse reactions in patients who received ADA or TOF as first-line b/tsDMARD therapy. Follow-up was limited, and physicians’ discretion to record adverse reactions limit the conclusions that can be drawn from these data. As more data on these risks become available, the safety of these drugs will be better understood.^38^

### Limitations

As an analysis of observational data, the challenges of missing outcomes data and nonrandomized treatment assignment were possible limitations to this study that may impact interpretation of the generalizability of the results. Our analysis relied on some assumptions, and violation of these would limit the reliability of the results. We assumed the joint counts, CRP levels, and patient global scores were missing at random (ie, whether an outcome was missing was not related to its value after conditioning on the observed data). Under this assumption, the missing data on joint counts, CRP levels, and patient global scores could be accounted for in the imputation model by the characteristics of the patients, the treating rheumatologists, and the clinics, which were fully observed, and accounting for these variables could produce unbiased results in the analysis. Availability of nursing staff time and other characteristics of the clinics and individual rheumatologists are plausible variables to explain whether a rheumatologist in a busy clinic would be able to prioritize recording complete data on all outcomes during a consultation with a patient. Nevertheless, we acknowledge that there may have been bias that could not be addressed if there were further unmeasured sources of missing data that were not accounted for in the imputation model.

The assumptions for inferring a causal treatment effect include that (1) the probability of initiating treatment with ADA or TOF may have depended on the measured baseline characteristics but did not depend on future disease activity at follow-up time points, (2) the probability of treatment assignment did not equal 0, and (3) a patient's observed DAS28-CRP at follow-up was the same as that patient's potential DAS28-CRP at follow-up after they followed the treatment that they were observed to be assigned to.^39,40^ It is possible that assignment to ADA or TOF was confounded by unmeasured characteristics that were not accounted for in this analysis. E-values for the ATE suggest that these results may be sensitive to unmeasured confounding (eResults 7 in Supplement 1). Although these assumptions cannot be verified, there was consistency between our analysis and other comparisons of ADA or TNFi therapy with TOF.^2,5^ This analysis emulated an intention-to-treat effect and not a per-protocol effect. Future work may address possible effects of patients stopping treatment between the 3- and 9-month time points.

## Conclusions

In this comparative effectiveness study, DAS28-CRP was significantly lower at 3 months for patients treated with TOF compared with ADA. However, 3 months of treatment with either drug led to substantive reductions in mean DAS28-CRP, consistent with remission. There was no difference in DAS28-CRP between patients receiving TOF or ADA at 9 months. The results of this observational study are consistent with clinical trial data^1,2^ and support the current EULAR treatment guidelines. The analysis serves as an exemplar of the TTE framework applied to an incomplete RWD.

## Notes

Supplement 1. **eMethods 1. **Protocol for the Target Trial to be Emulated **eMethods 2.** Description of the OPAL Dataset **eMethods 3.** Directed Acyclic Diagram **eMethods 4.** Overview of the Analytical Procedure **eMethods 5.** Multiple Imputation **eMethods 6.** Stable Balancing Weights **eMethods 7.** Bootstrapping Procedure **eResults 1.** Performance of Multiple Imputation **eResults 2.** Properties of Stable Balancing Weights **eResults 3.** Balance Checks **eResults 4.** Weighted Components of the DAS28CRP **eResults 5.** Point Estimates **eResults 6.** Drug Cessations Due to Adverse Reactions **eResults 7.** E-Values to Quantify Sensitivity of Results to Possible Unmeasured Confounding **eAppendix.** Code
eReferences

Supplement 2.
OPAL Rheumatology Network

Supplement 3.
Data Sharing Statement
