# Supplementary material for: Comparative Effectiveness of Adalimumab vs Tofacitinib in Patients With Rheumatoid Arthritis in Australia
Source: JAMA Netw Open. 2023 Jun 29;6(6):e2320851. doi: 10.1001/jamanetworkopen.2023.20851 (PMC10311390; doi:10.1001/jamanetworkopen.2023.20851)
Supplement: Supplement 1. — eMethods 1. Protocol for the Target Trial to be Emulated eMethods 2. Description of the OPAL Dataset eMethods 3. Directed Acyclic Diagram eMethods 4. Overview of the Analytical Procedure eMethods 5. Multiple Imputation eMethods 6. Stable Balancing Weights eMethods 7. Bootstrapping Procedure eResults 1. Performance of Multiple Imputation eResults 2. Properties of Stable Balancing Weights eResults 3. Balance Checks eResults 4. Weighted Components of the DAS28CRP eResults 5. Point Estimates eResults 6. Drug Cessations Due to Adverse Reactions eResults 7. E-Values to Quantify Sensitivity of Results to Possible Unmeasured Confounding eAppendix. Code eReferences [file jamanetwopen-e2320851-s001.pdf]

Supplemental Online Content

Deakin CT, De Stavola BL, Littlejohn G, et al; OPAL Rheumatology Network. Comparative effectiveness of adalimumab vs tofacitinib in patients with rheumatoid arthritis in Australia. *JAMA Netw Open.* 2023;6(6):e2320851. doi:10.1001/jamanetworkopen.2023.20851

eMethods 1. Protocol for the Target Trial to be Emulated

eMethods 2. Description of the OPAL Dataset

eMethods 3. Directed Acyclic Diagram

eMethods 4. Overview of the Analytical Procedure

eMethods 5. Multiple Imputation

eMethods 6. Stable Balancing Weights

eMethods 7. Bootstrapping Procedure

eResults 1. Performance of Multiple Imputation

eResults 2. Properties of Stable Balancing Weights

eResults 3. Balance Checks

eResults 4. Weighted Components of the DAS28CRP

eResults 5. Point Estimates

eResults 6. Drug Cessations Due to Adverse Reactions

eResults 7. E-Values to Quantify Sensitivity of Results to Possible Unmeasured Confounding

eAppendix. Code

eReferences.

This supplemental material has been provided by the authors to give readers additional information about their work.

© 2023 Deakin CT et al. *JAMA Network Open.*

eMethods 1: Protocol for the Target Trial to be Emulated

The aim of this study was to estimate the average treatment effect (ATE) of tofacitinib (TOF) compared to adalimumab (ADA) at 3 and 9 months after initiating treatment in patients with rheumatoid arthritis (RA) who are new users of a biologic or targeted synthetic disease modifying anti-rheumatic drug (b/tsDMARD). The ATE was defined as the difference in mean disease activity score, Disease Activity Score-28 with C-reactive protein (DAS28CRP), a composite disease activity score which includes swollen and tender joint counts, a systemic inflammatory marker and a patient-reported outcome. This objective is equivalent to aiming to estimate the intention-to-treat effect in a randomised controlled trial.

eTable 1 lists the details of the pre-specified protocol for the target trial which was developed prior to analysis [1].

eTable 1. Design features of pre-specified protocol for the target trial of ADA versus TOF to be emulated

Design feature	Detail
Eligibility criteria	Adult patients diagnosed with RA whose first recorded visit occurred between 1 April 2015 and 1 January 2021 with no prior recorded b/tsDMARD and at least 6 months from their first-recorded visit until Time Zero and at least 6 months of treatment with a conventional synthetic DMARD (csDMARD) immediately prior to Time Zero.
Treatment strategies	Initiate treatment with either ADA (2.9 mg daily) or TOF (10 mg daily). Continue treatment during follow-up unless the patient experiences an adverse event or contra-indication.
Assignment procedures	Participants are randomised to either treatment strategy and are unblinded as to the treatment they receive i.e. a pragmatic trial
Follow-up period	Starts at randomisation (baseline), with follow-up visits at 3 months and 9 months as per the government reimbursement schedule.
Outcomes	Difference in DAS28CRP at 3 months and 9 months.
Causal contrasts of interest	Intention-to-treat effects
Analysis plan	Intention-to-treat effect estimated by comparing mean DAS28CRP at follow-up in patients assigned to treatment with ADA or TOF, with adjustment for baseline DAS28CRP.
Time zero	Baseline/Time Zero defined as the date at which patients are prescribed ADA or TOF as their first-recorded b/tsDMARD biologic medication. This definition ensures eligibility criteria can only be met at a single time.

© 2023 Deakin CT et al. *JAMA Network Open.*

The eligibility criteria were intended to select (or at least enrich for) patients with RA who are new users of a b/tsDMARD, within the constraints of what is feasible for a real-world dataset. Patients were required to have at least one component of the DAS28CRP recorded at baseline or follow-up (3 months or 9 months).

The beginning of the study window is based on when both drugs were available in Australia (TOF became available in October 2015, new users would have required at least 6 months of prior csDMARD treatment). Since biosimilars for ADA only became available in April 2021, patients who were new users of an ADA biosimilar would not yet have had a full 3 months of follow-up when data were extracted (5 July 2021), and so were excluded from analysis.

© 2023 Deakin CT et al. *JAMA Network Open.*

eMethods 2: Description of the OPAL Dataset

The OPAL dataset is a large collection of real-world clinical data on 216,138 patients with rheumatic diseases treated by 112 rheumatologists at 43 clinics around Australia who have agreed to share their electronic medical records (EMR) [2]. The rheumatologists are predominantly in private practice community clinics, and this reflects how patients are managed in Australia. Data are entered at the point of care into customised EMR, Audit4 (Software 4 Specialists Pty Ltd). The 112 rheumatologists represent approximately one third of rheumatologists in Australia.

At quarterly intervals, data are deidentified while on the clinicians' individual servers, and are then extracted and aggregated for research purposes. The dataset includes information on demographics, disease history, disease activity measures, comorbidities, pathology and medication use, as well as patient-reported outcomes and characteristics of the rheumatologists.

Patients consent to involvement via an opt-out consent model. Ethical approval for OPAL's research activities using de-identified data has been provided by the University of New South Wales Human Research Ethics Committee (HC17799). Under this approval, data cannot be provided for patients aged under 18 years, or patients who cannot withdraw consent or patients who are potentially identifiable, such as those who have died or those aged over 95 years. Ethical approval was also obtained for this specific project (HC210647).

Visit frequency occurs mostly according to clinical need but also at the frequency required by government reimbursement criteria for biologic and targeted synthetic disease-modifying anti-rheumatic drugs (b/tsDMARDs). Under the reimbursement criteria, an initial application is made when a patient is initiated on a b/tsDMARD, and then response must be assessed at 3 months, and then at 6-monthly intervals. These applications require data on the swollen joint count-28 (SJC28), tender joint count-28 (TJC28), C-reactive protein (CRP) and erythrocyte sedimentation rate (ESR). The EMR software can automatically generate electronic reimbursement applications after the rheumatologist enters these data into the EMR, and this can be a time-saving incentive to enter joint counts. Pathology data are automatically downloaded from the pathology laboratories into the EMR.

Additional outcomes recorded in the EMR include the physician's global score, the patient's global score, the patient's pain score and composite disease activity scores such as the DAS28CRP.

The rheumatologist has discretion to record additional clinical data such as comorbidities and medication history in the EMR. Consequently, it is possible that comorbidities and certain medications are under-recorded.

Uniquely in the Australian setting, the sequence or class of b/tsDMARDs is not specified by the government. Once a patient qualifies for a b/tsDMARD, their rheumatologist can choose which b/tsDMARD is the most suited to their clinical needs.

To maintain the de-identification of the data, no identifiers for individual practitioners or clinics are provided. Consequently, random effects cannot be used to account for these levels of

© 2023 Deakin CT et al. *JAMA Network Open.*

clustering in the data. Practitioner effects were accounted for by using practitioner features, including their gender, years of experience, overall tendency to prescribe b/tsDMARDs and overall tendency to record the patient's global score.

Similarly, categories for certain variables were combined to preserve patient de-identification. For example, there were fewer clinics in Queensland and Western Australia participating in OPAL, and so these two states are combined.

Disease activity variables

Data on the 4 components of the composite disease activity outcome of interest, DAS28CRP, were collected at baseline, at 3 months and at 9 months i.e. SJC28, TJC28, the patient global score and and the CRP. The physician global score and the ESR were also collected as these measures were thought to contain further information about disease activity that could be exploited during imputation of the missing DAS28CRP components.

To minimise missing data and allow for visits that occurred slightly outside of the follow-up visit schedule required for government reimbursement, windows of time around Time Zero and the 3 month and 9 month follow-up timepoints were allowed. For Time Zero, this was -3.0 months up to and including 1.5 months. For 3 months, this was 1.6 months up to and including 6.0 months. For 9 months, this was 6.1 months up to and including 12.0 months. If more than one visit was identified within these windows, then the visit with the least missing outcomes data that occurred closest to the expected date for that timepoint was selected.

The distributions of timepoints used were similar across the treatment groups for Time Zero, 3 months and 9 months in eFigure 1, which shows that timepoints of visits used were close to the expected follow-up schedule and similar by treatment group.

© 2023 Deakin CT et al. *JAMA Network Open.*

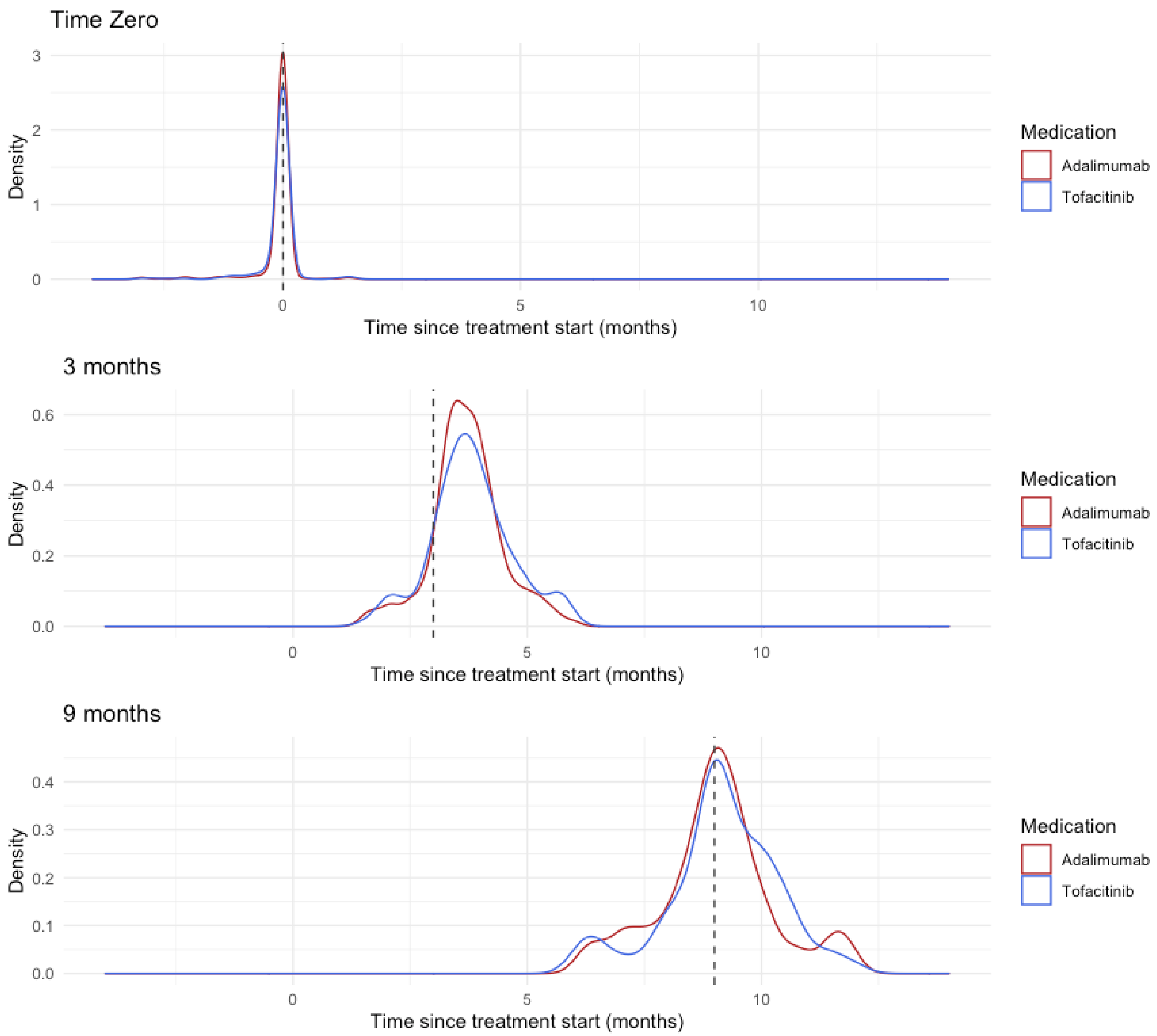

eFigure 1: Distribution of timepoints used by treatment group.

The median [interquartile range (IQR)] for Time Zero in the ADA group was 0 [0, 0] months, while the median [IQR] for Time Zero in the TOF group was 0 [0, 0] months.

The median [interquartile range (IQR)] for the 3 month timepoint in the ADA group was 3.7 [3.3, 4.1] months, while the median [IQR] for the 3 month timepoint in the TOF group was 3.7 [3.3, 4.3] months.

The median [interquartile range (IQR)] for the 9 month timepoint in the ADA group was 9.1 [8.5, 9.6] months, while the median [IQR] for the 9 month timepoint in the TOF group was 9.2 [8.7, 9.9] months.

The 3 month and 9 month timepoints were well within crude Kaplan-Meier estimates of median treatment persistence for ADA and TOF, at 21 (95% CI 15.7, 25.5) months for ADA and 32.5 (95% CI 24.4, 45.5) months for TOF. Note that these crude estimates have not been weighted

© 2023 Deakin CT et al. *JAMA Network Open.*

to account for baseline differences between treatment groups and should not be interpreted as an analysis of treatment persistence.

There were 560 patients (98.4%) remaining on ADA and 264 patients (96.7%) remaining on TOF at 3 months.

There were 304 patients (53.4%) remaining on ADA and 173 patients (63.4%) remaining on TOF at 9 months.

Of the 842 included patients who initiated treatment, 56 (7%) who initiated ADA and 14 (2%) who initiated TOF were lost to follow-up after the 3 month timepoint. There were no patients who were lost to follow-up due to mortality.

Baseline covariates

Variable type and overall completeness for the baseline covariates used in this analysis are described in eTable 2. While data on additional recorded comorbidities at baseline were also collected, including previous cardiac disease, cancer, diabetes, tuberculosis, lung disease, thromboembolism and clotting disorder, these comorbidities were not used in the analysis due to sparseness.

eTable 2. Description of baseline covariates in the OPAL dataset

Covariate	Type of variable	Completeness	Category
Age	Numeric	100%	-
Gender	Binary categorical	99.2%	Female
Gender	Binary categorical	99.2%	Male
State	Categorical	99.8%	Victoria
State	Categorical	99.8%	New South Wales
State	Categorical	99.8%	Australian Capital Territory
State	Categorical	99.8%	Tasmania
State	Categorical	99.8%	Queensland and Western Australia
Regional location	Categorical	99.9%	Major cities
Regional location	Categorical	99.9%	Inner regional

© 2023 Deakin CT et al. *JAMA Network Open.*

Covariate	Type of variable	Completeness	Category
Regional location	Categorical	99.9%	Outer regional and remote
Disease duration recorded in Audit4	Numeric	100%	-
Year of treatment start	Ordered categorical	100%	2015 and 2016
Year of treatment start	Ordered categorical	100%	2017
Year of treatment start	Ordered categorical	100%	2018
Year of treatment start	Ordered categorical	100%	2019
Year of treatment start	Ordered categorical	100%	2020
Year of treatment start	Ordered categorical	100%	2021
DAS28CRP	Numeric	60%	-
Nurse at clinic	Binary categorical	100%	Yes
Nurse at clinic	Binary categorical	100%	No
Practitioner gender	Binary categorical	100%	Female
Practitioner gender	Binary categorical	100%	Male
Practitioner experience	Categorical	100%	0-15 years
Practitioner experience	Categorical	100%	16-30 years
Practitioner experience	Categorical	100%	Over 30 years
Practitioner overall tendency to complete patient global score	Binary categorical	100%	0%-75% complete
Practitioner overall tendency to complete patient global score	Binary categorical	100%	76%-100% complete
Practitioner overall tendency to prescribe b/tsDMARDs	Binary categorical	100%	0%-25% of total prescriptions

© 2023 Deakin CT et al. *JAMA Network Open.*

Covariate	Type of variable	Completeness	Category
Practitioner overall tendency to prescribe b/tsDMARDs	Binary categorical	100%	26%-100% of total prescriptions
Prior renal disease	Binary categorical	100%	Yes
Prior renal disease	Binary categorical	100%	No
Prior methotrexate	Binary categorical	100%	Yes
Prior methotrexate	Binary categorical	100%	No
Prior hydroxychloroquine	Binary categorical	100%	Yes
Prior hydroxychloroquine	Binary categorical	100%	No
Prior leflunomide	Binary categorical	100%	Yes
Prior leflunomide	Binary categorical	100%	No
Prior sulfasalazine	Binary categorical	100%	Yes
Prior sulfasalazine	Binary categorical	100%	No
Prior oral corticosteroids	Binary categorical	100%	Yes
Prior oral corticosteroids	Binary categorical	100%	No
Concomitant methotrexate	Binary categorical	100%	Yes
Concomitant methotrexate	Binary categorical	100%	No
Concomitant hydroxychloroquine	Binary categorical	100%	Yes
Concomitant hydroxychloroquine	Binary categorical	100%	No

© 2023 Deakin CT et al. *JAMA Network Open.*

Covariate	Type of variable	Completeness	Category
Concomitant leflunomide	Binary categorical	100%	Yes
Concomitant leflunomide	Binary categorical	100%	No
Concomitant sulfasalazine	Binary categorical	100%	Yes
Concomitant sulfasalazine	Binary categorical	100%	No
Concomitant oral corticosteroids	Binary categorical	100%	Yes
Concomitant oral corticosteroids	Binary categorical	100%	No

Dummy variables were generated during data preparation to represent all categorical and ordered categorical variables, as these were required in later steps of the analysis.

The recording of the presence of a comorbidity in the EMR requires an active step by the rheumatologist. Consequently, the variable on prior recorded renal disease is best interpreted as “known recorded prior renal disease” or “no recorded prior renal disease or prior renal disease unknown”. The nature of how comorbidities are recorded may account for the sparseness of the other comorbidities that were considered as possible relevant prior comorbidities.

Characteristics of patients excluded due to having no disease activity data

The demographic and clinical features of patients who were excluded due to having no components of DAS28CRP recorded at baseline, 3 months and 9 months are described in eTable 3, along with those features for the included cohort.

eTable 3. Baseline features of included and excluded patients

Feature	Category	Complete data (Included / Excluded)	Included patients (n=842)	Excluded patients (n=326)
Treatment group	Adalimumab	100% / 100%	569 (67.6%)	170 (52.1%)
Treatment group	Tofacitinib	100% / 100%	273 (32.4%)	156 (47.9%)
Age	-	100% / 100%	57 [48, 66]; 18 - 88	59 [48, 67]; 16 - 86
Gender	Female	99.2% / 100%	595 (70.7%)	229 (70.2%)
Gender	Male	99.2% / 100%	247 (29.3%)	97 (29.8%)
State	Victoria	99.8% / 100%	361 (42.9%)	125 (38.3%)
State	New South Wales	99.8% / 100%	257 (30.5%)	65 (19.9%)
State	Australian Capital Territory	99.8% / 100%	67 (8%)	23 (7.1%)
State	Tasmania	99.8% / 100%	42 (5%)	11 (3.4%)
State	Queensland and Western Australia	99.8% / 100%	113 (13.4%)	102 (31.3%)
Regional location	Major cities	99.9% / 100%	520 (61.8%)	177 (54.3%)
Regional location	Inner regional	99.9% / 100%	234 (27.8%)	111 (34%)
Regional location	Outer regional and remote	99.9% / 100%	87 (10.3%)	38 (11.7%)
Disease duration recorded in Audit4	-	100% / 100%	1.1 [0.7, 1.8]; 0.5 - 5.6	1 [0.7, 1.8]; 0.5 - 5.1
Year of treatment start	2015 and 2016	100% / 100%	93 (11%)	39 (12%)

© 2023 Deakin CT et al. *JAMA Network Open.*

Feature	Category	Complete data (Included / Excluded)	Included patients (n=842)	Excluded patients (n=326)
Year of treatment start	2017	100% / 100%	170 (20.2%)	56 (17.2%)
Year of treatment start	2018	100% / 100%	177 (21%)	68 (20.9%)
Year of treatment start	2019	100% / 100%	183 (21.7%)	79 (24.2%)
Year of treatment start	2020	100% / 100%	180 (21.4%)	61 (18.7%)
Year of treatment start	2021	100% / 100%	39 (4.6%)	23 (7.1%)
Nurse at clinic	Yes	100% / 100%	358 (42.5%)	103 (31.6%)
Nurse at clinic	No	100% / 100%	484 (57.5%)	223 (68.4%)
Practitioner gender	Female	100% / 100%	347 (41.2%)	135 (41.4%)
Practitioner gender	Male	100% / 100%	495 (58.8%)	191 (58.6%)
Practitioner experience	0-15 years	100% / 100%	261 (31%)	125 (38.3%)
Practitioner experience	16-30 years	100% / 100%	368 (43.7%)	70 (21.5%)
Practitioner experience	Over 30 years	100% / 100%	213 (25.3%)	131 (40.2%)
Practitioner overall tendency to complete patient global score	0%-75% complete	100% / 100%	345 (41%)	245 (75.2%)
Practitioner overall tendency to complete patient global score	76%-100% complete	100% / 100%	497 (59%)	81 (24.8%)
Practitioner overall tendency to prescribe b/tsDMARDs	0%-25% of total prescriptions	100% / 100%	710 (84.3%)	198 (60.7%)
Practitioner overall tendency to prescribe b/tsDMARDs	26-100% of total prescriptions	100% / 100%	132 (15.7%)	128 (39.3%)

© 2023 Deakin CT et al. *JAMA Network Open.*

Feature	Category	Complete data (Included / Excluded)	Included patients (n=842)	Excluded patients (n=326)
Prior renal disease	Yes	100% / 100%	359 (42.6%)	142 (43.6%)
Prior renal disease	No	100% / 100%	483 (57.4%)	184 (56.4%)
Prior methotrexate	Yes	100% / 100%	539 (64%)	199 (61%)
Prior methotrexate	No	100% / 100%	303 (36%)	127 (39%)
Prior hydroxychloroquine	Yes	100% / 100%	334 (39.7%)	152 (46.6%)
Prior hydroxychloroquine	No	100% / 100%	508 (60.3%)	174 (53.4%)
Prior leflunomide	Yes	100% / 100%	256 (30.4%)	72 (22.1%)
Prior leflunomide	No	100% / 100%	586 (69.6%)	254 (77.9%)
Prior sulfasalazine	Yes	100% / 100%	232 (27.6%)	56 (17.2%)
Prior sulfasalazine	No	100% / 100%	610 (72.4%)	270 (82.8%)
Prior oral corticosteroids	Yes	100% / 100%	174 (20.7%)	174 (53.4%)
Prior oral corticosteroids	No	100% / 100%	668 (79.3%)	152 (46.6%)
Concomitant methotrexate	Yes	100% / 100%	230 (27.3%)	107 (32.8%)
Concomitant methotrexate	No	100% / 100%	612 (72.7%)	219 (67.2%)
Concomitant hydroxychloroquine	Yes	100% / 100%	202 (24%)	87 (26.7%)
Concomitant hydroxychloroquine	No	100% / 100%	640 (76%)	239 (73.3%)
Concomitant leflunomide	Yes	100% / 100%	150 (17.8%)	47 (14.4%)
Concomitant leflunomide	No	100% / 100%	692 (82.2%)	279 (85.6%)

© 2023 Deakin CT et al. *JAMA Network Open.*

Feature	Category	Complete data (Included / Excluded)	Included patients (n=842)	Excluded patients (n=326)
Concomitant sulfasalazine	Yes	100% / 100%	128 (15.2%)	26 (8%)
Concomitant sulfasalazine	No	100% / 100%	714 (84.8%)	300 (92%)
Concomitant oral corticosteroids	Yes	100% / 100%	126 (15%)	159 (48.8%)
Concomitant oral corticosteroids	No	100% / 100%	716 (85%)	167 (51.2%)

© 2023 Deakin CT et al. *JAMA Network Open.*

Of the included patients, 11 (1%) in the ADA group and 4 (0%) in the TOF group were aged over 50 years and had prior cardiovascular disease.

Characteristics of complete cases

eTable 4 describes the features of the 292 patients and 261 patients with all components of the DAS28CRP recorded at 0 months and 3 months, and at 0 months and 9 months, respectively, along with the features of the included cohort.

Given the small proportion of patients with sufficient complete data for estimating the ATE at 3 and 9 months (34.7% and 31%, respectively) and the presence of differences in certain baseline characteristics for these complete cases (e.g. more patients with prior renal disease and differences in prior and concomitant medication), a complete case analysis was considered to be uninformative.

eTable 4. Baseline features of included patients and complete cases

Feature	Category	Included patients (n=842)	Patients with complete DAS28CRP at 0 and 3 months (n= 292)	Patients with complete DAS28CRP at 0 and 9 months (n= 261)
Treatment group	Adalimumab	569 (67.6%)	202 (69.2%)	173 (66.3%)
	Tofacitinib	273 (32.4%)	90 (30.8%)	88 (33.7%)
Age	-	57 [48, 66]; 18 - 88	59 [50, 67]; 18 - 88	58 [50, 65]; 18 - 86
Gender	Female	595 (70.7%)	213 (72.9%)	184 (70.5%)
	Male	247 (29.3%)	79 (27.1%)	77 (29.5%)
State	Victoria	361 (42.9%)	129 (44.2%)	116 (44.4%)
	New South Wales	257 (30.5%)	86 (29.5%)	77 (29.5%)

© 2023 Deakin CT et al. *JAMA Network Open.*

Feature	Category	Included patients (n=842)	Patients with complete DAS28CRP at 0 and 3 months (n= 292)	Patients with complete DAS28CRP at 0 and 9 months (n= 261)
	Australian Capital Territory	67 (8%)	24 (8.2%)	19 (7.3%)
	Tasmania	42 (5%)	5 (1.7%)	6 (2.3%)
	Queensland and Western Australia	113 (13.4%)	48 (16.4%)	43 (16.5%)
Regional location	Major cities	520 (61.8%)	195 (66.8%)	169 (64.8%)
	Inner regional	234 (27.8%)	73 (25%)	70 (26.8%)
	Outer regional and remote	87 (10.3%)	24 (8.2%)	22 (8.4%)
Disease duration recorded in Audit4	-	1.1 [0.7, 1.8]; 0.5 - 5.6	1.1 [0.8, 1.8]; 0.5 - 5	1.1 [0.7, 1.8]; 0.5 - 4.9
Year of treatment start	2015 and 2016	93 (11%)	36 (12.3%)	39 (14.9%)
	2017	170 (20.2%)	77 (26.4%)	67 (25.7%)
	2018	177 (21%)	68 (23.3%)	59 (22.6%)
	2019	183 (21.7%)	64 (21.9%)	61 (23.4%)
	2020	180 (21.4%)	41 (14%)	35 (13.4%)
	2021	39 (4.6%)	6 (2.1%)	0 (0%)
Nurse at clinic	Yes	358 (42.5%)	124 (42.5%)	100 (38.3%)

© 2023 Deakin CT et al. *JAMA Network Open.*

Feature	Category	Included patients (n=842)	Patients with complete DAS28CRP at 0 and 3 months (n= 292)	Patients with complete DAS28CRP at 0 and 9 months (n= 261)
	No	484 (57.5%)	168 (57.5%)	161 (61.7%)
Practitioner gender	Female	347 (41.2%)	89 (30.5%)	80 (30.7%)
	Male	495 (58.8%)	203 (69.5%)	181 (69.3%)
Practitioner experience	0-15 years	261 (31%)	65 (22.3%)	54 (20.7%)
	16-30 years	368 (43.7%)	139 (47.6%)	128 (49%)
	Over 30 years	213 (25.3%)	88 (30.1%)	79 (30.3%)
Practitioner overall tendency to complete patient global score	0%-75% complete	345 (41%)	48 (16.4%)	37 (14.2%)
	76%-100% complete	497 (59%)	244 (83.6%)	224 (85.8%)
Practitioner overall tendency to prescribe b/tsDMARDs	0%-25% of total prescriptions	710 (84.3%)	253 (86.6%)	230 (88.1%)
	26-100% of total prescriptions	132 (15.7%)	39 (13.4%)	31 (11.9%)
Prior renal disease	Yes	359 (42.6%)	172 (58.9%)	125 (47.9%)
	No	483 (57.4%)	120 (41.1%)	136 (52.1%)
Prior methotrexate	Yes	539 (64%)	252 (86.3%)	197 (75.5%)
	No	303 (36%)	40 (13.7%)	64 (24.5%)
Prior hydroxychloroquine	Yes	334 (39.7%)	158 (54.1%)	127 (48.7%)
	No	508 (60.3%)	134 (45.9%)	134 (51.3%)

© 2023 Deakin CT et al. *JAMA Network Open.*

Feature	Category	Included patients (n=842)	Patients with complete DAS28CRP at 0 and 3 months (n= 292)	Patients with complete DAS28CRP at 0 and 9 months (n= 261)
Prior leflunomide	Yes	256 (30.4%)	105 (36%)	80 (30.7%)
	No	586 (69.6%)	187 (64%)	181 (69.3%)
Prior sulfasalazine	Yes	232 (27.6%)	118 (40.4%)	90 (34.5%)
	No	610 (72.4%)	174 (59.6%)	171 (65.5%)
Prior oral corticosteroids	Yes	174 (20.7%)	230 (78.8%)	176 (67.4%)
	No	668 (79.3%)	62 (21.2%)	85 (32.6%)
Concomitant methotrexate	Yes	230 (27.3%)	97 (33.2%)	93 (35.6%)
	No	612 (72.7%)	195 (66.8%)	168 (64.4%)
Concomitant hydroxychloroquine	Yes	202 (24%)	103 (35.3%)	81 (31%)
	No	640 (76%)	189 (64.7%)	180 (69%)
Concomitant leflunomide	Yes	150 (17.8%)	64 (21.9%)	47 (18%)
	No	692 (82.2%)	228 (78.1%)	214 (82%)
Concomitant sulfasalazine	Yes	128 (15.2%)	69 (23.6%)	50 (19.2%)
	No	714 (84.8%)	223 (76.4%)	211 (80.8%)
Concomitant oral corticosteroids	Yes	126 (15%)	200 (68.5%)	150 (57.5%)
	No	716 (85%)	92 (31.5%)	111 (42.5%)

© 2023 Deakin CT et al. *JAMA Network Open.*

eMethods 3: Directed Acyclic Diagram

eFigure 2 is a directed acyclic diagram (DAG) showing putative relationships between measured and unmeasured covariates, and the exposure and outcome. Each arrow in the DAG represents a relationship that is assumed to be causal for the purpose of the analysis. These putative relationships were discussed prior to the analysis.

The intention of a DAG is not to depict these relationships with absolute certainty, as this cannot be known, but rather to give transparency about the *a priori* assumptions underpinning the causal relationships between exposure and outcomes, and possible confounders, and to inform selection of variables to be included or excluded from propensity score models [3].

For example, a clinician's experience may be influenced by their training (which is unmeasured) and may also be related to their gender as a higher proportion of rheumatologists with over 30 years of experience are male. Experience is assumed to directly influence disease activity (the outcome) and the treatment prescribed (exposure). However, indirect influences on treatment and disease activity are also assumed for the clinician's experience e.g. via the presence of a nurse at their practice, the patient's previous disease activity and previously prescribed immunosuppressant medication.

DAGs were generated and analysed using the `dagitty` package [4], and were visualised using the `ggdag` package [5].

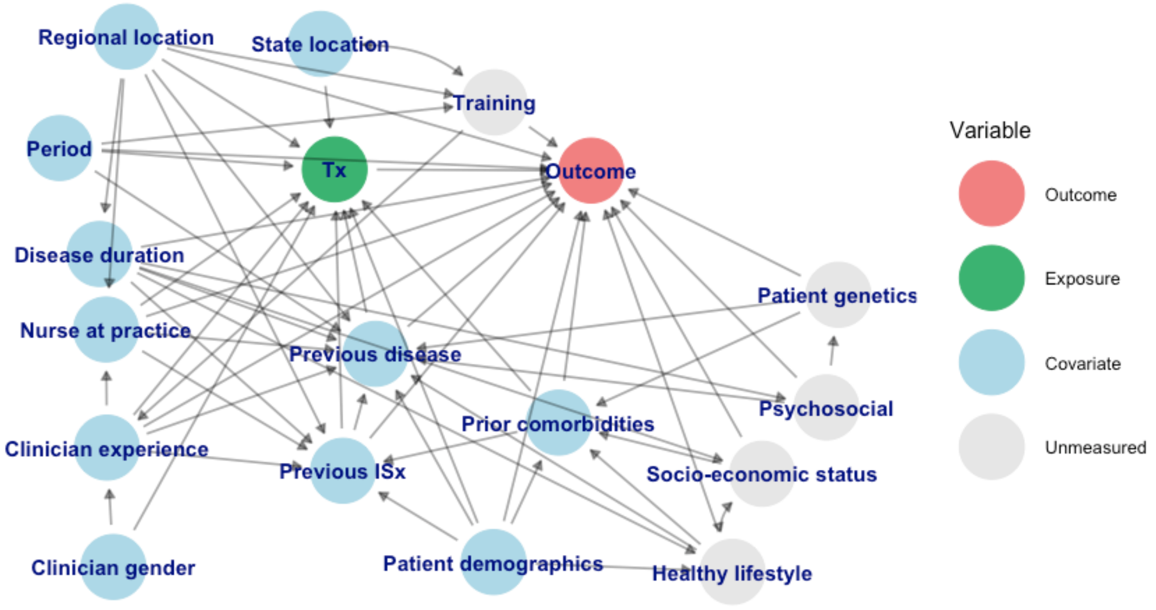

eFigure 2: Relationships between exposure (treatment), measured and unmeasured covariates and outcomes depicting different variable types. ISx, immunosuppression; Tx, treatment.

© 2023 Deakin CT et al. *JAMA Network Open.*

The DAG can be used to identify colliders, which are variables that have exposure and outcome as a common cause, as well as confounders, which are a common cause of exposure and outcome. The propensity score model should control for the minimum set of confounders in order to reduce bias, but not control for any colliders as this would induce bias.

Possible colliders were identified in eFigure 3 using the `ggdag_collider` function.

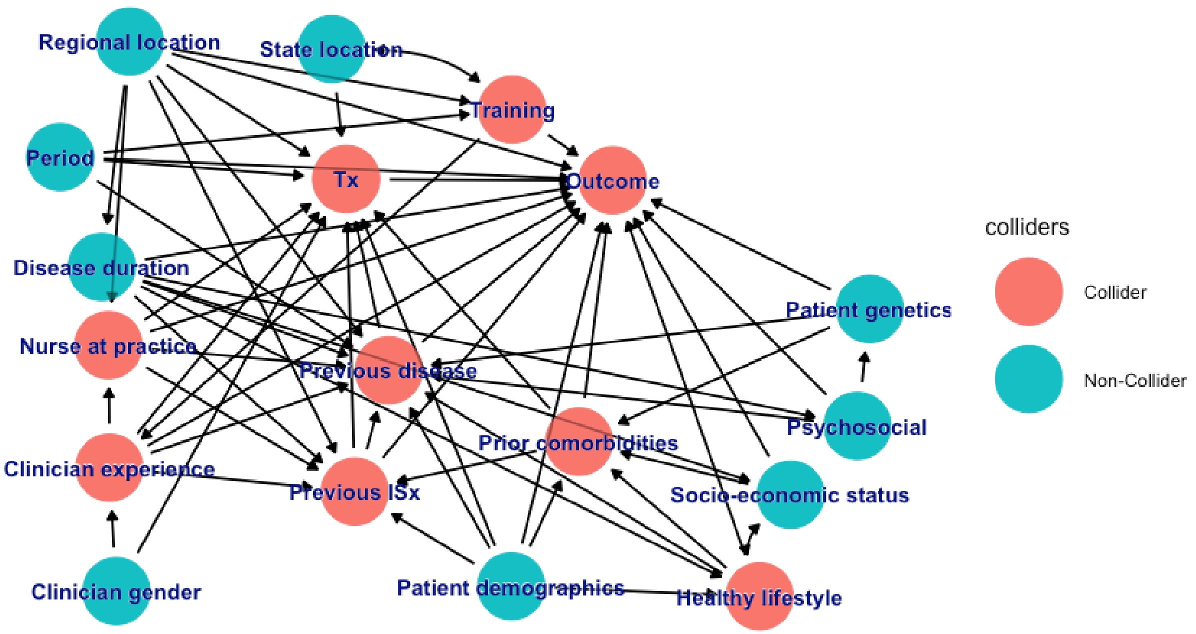

eFigure 3: Identification of possible colliders in the causal pathway. ISx, immunosuppression; Tx, treatment.

eFigure 4 shows the minimum adjustment set for the measured covariates identified using `ggdag_adjustment_set`. Note that this set also accounts for the effects of possible collider variables. The minimum adjustment set is based on the arrows in eFigure 2 and is intended to identify the smallest number of potential confounding variables to adjust for in the propensity score model while avoiding collider bias. Note that disease duration, which is a common cause of both the outcome (directly and via indirect pathways) and the exposure (indirect pathways via previous immunosuppression or previous disease activity), does not need to be adjusted for in the minimal set, but adjusting for it will not introduce collider bias.

© 2023 Deakin CT et al. *JAMA Network Open.*

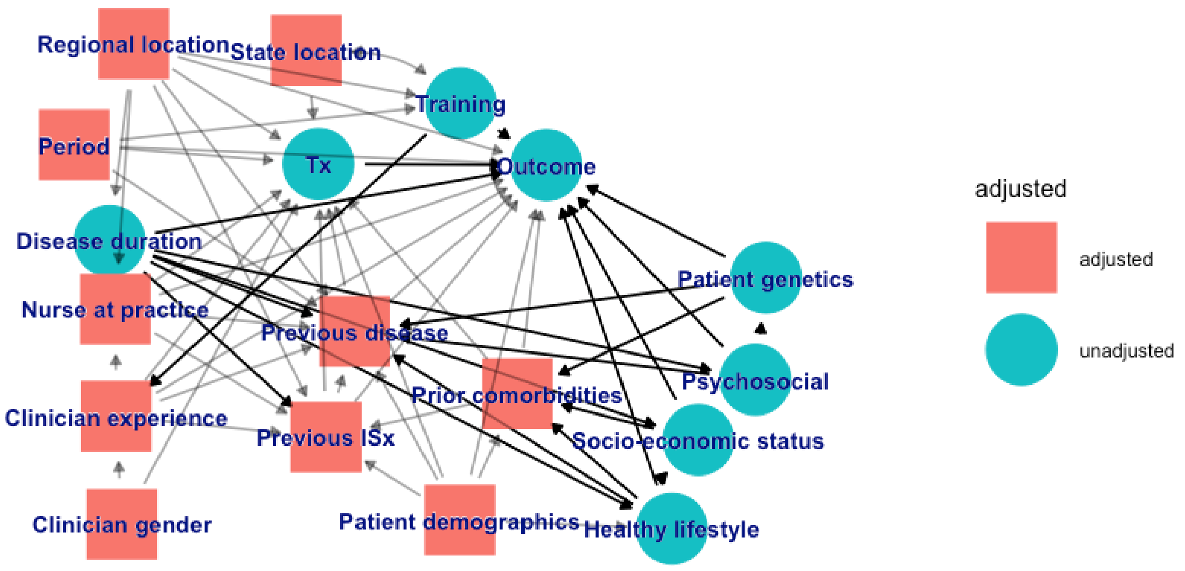

eFigure 4: Minimum adjustment set for measured covariates. ISx, immunosuppression; Tx, treatment.

© 2023 Deakin CT et al. *JAMA Network Open.*

eMethods 4: Overview of the Analytical Procedure

Definitions

- Y_0 represents the components of DAS28CRP at time 0
- Y_1 represents the components of DAS28CRP at time 3
- Y_2 represents the components of DAS28CRP at time 9
- S is an indicator for whether *any* of Y_1 or Y_2 are observed i.e. whether the patient has any DAS28CRP components at follow-up (n=699 have $S = 1$)
- T is the treatment indicator, $T = 1$ for TOF (n=273), $T = 0$ for ADA (n=569)
- X represents fully observed variables at time 0, including:
  - Patient demographics (age, gender, state, regional location, disease duration)
  - Year of treatment start
  - Prior/concomitant medication
  - Prior/concomitant comorbidities
  - Practitioner gender, experience, tendency to prescribe biologics
  - Availability of nurse at practice, regional location of practice
  - An indicator for each individual practitioner's overall tendency to record the patient global based on their entire EMR

The sample comprises any individuals with an observed Y_0, Y_0, or Y_2 (n=842).

As percentages of missing data for variables in X were below 1%, missing data were replaced by the most frequent category.

Assumptions

Let $Y_1^{(0)}$, $Y_2^{(0)}$, $Y_1^{(1)}$ and $Y_2^{(1)}$ denote the potential outcomes at 3 months and 9 months follow-up.

1. (X, T, Y_0, Y_1, Y_2) is missing at random (MAR) i.e. any missing data for any DAS28CRP components at any timepoint are missing at random

2. $P(T = 1|X, Y_0, Y_1^{(0)}, Y_2^{(0)}, Y_1^{(1)}, Y_2^{(1)}, S = 1) = P(T = 1|X, Y_0, S = 1)$ i.e. the probability of treatment assignment depends on the baseline covariates and is not affected by potential outcomes at 3 months or at 9 months

3. Stable unit treatment value i.e. one patient's outcome is not affected by other patients' exposure to treatment

4. Positivity i.e. the conditional probability of patients receiving TOF given X and Y_0 does not equal zero or one for any value of X or Y_0

5. Consistency i.e. the potential outcome of a patient when assigned to that patient's observed exposure is the same as that patient's observed outcome

© 2023 Deakin CT et al. *JAMA Network Open.*

Methodology

This methodology uses random forest multiple imputation (RF-MI) to impute missing DAS28CRP [6], and stable balancing weights (SBW) to simultaneously account for treatment assignment ($T = 1$ for TOF, $T = 0$ for ADA) and whether patients have outcomes data at follow-up ($S = 1$ for at least one DAS28CRP component observed at 3 or at 9 months, $S = 0$ for no DAS28CRP components at either follow-up timepoint) [7].

Balancing is a different approach for generating weights compared to direct modelling approaches, such as propensity score models. Both approaches aim to achieve balanced distributions of covariates between different groups of a sample e.g. treatment groups. Whereas modelling approaches maximise the fit of a model for treatment assignment to then derive weights and imposes balance conditions on the covariates used to estimate the propensity score, a balancing approach directly optimises certain features of the weights and implicitly models the propensity score [8]. When the correct treatment assignment model is unknown, the balancing approach can result in better covariate balance with weights that are minimally dispersed and stable, meaning that they have minimum variance. This may reduce the standard error of the estimator.

The weights generated by SBW can be thought of as weights for a ternary (3-level) treatment consisting of:

1. Patients treated with ADA and whose outcomes are observed ($(T, S) = (0,1)$, n=464);
2. Patients treated with TOF and whose outcomes are observed ($(T, S) = (1,1)$, n=235); and
3. Patients who ‘drop out’ and their outcomes are not observed ($S = 0$, n=143).

The weights need to ensure that the individuals in the first two groups have the same marginal means of (X, Y_0) as the marginal means of (X, Y_0) in the entire sample i.e. all three groups (n=842).

After this methodology was used to generate the point estimates for ATE, the whole procedure was bootstrapped using $B = 1000$ bootstrap samples in order to generate a 95% confidence interval for the estimates using the MI boot percentile method [9].

Steps

To generate the point estimates for the ATE at 3 months and at 9 months:

1. RF-MI to generate $m = 10$ imputed datasets, followed by calculation of DAS28CRP from imputed DAS28CRP components

2. SBW to account for selection by $(T, S) = (0,1)$ i.e. to generate weights for patients on ADA to account for non-randomised treatment assignment and for whether outcomes were observed at follow-up

© 2023 Deakin CT et al. *JAMA Network Open.*

3. SBW to account for selection by $(T, S) = (1,1)$ i.e. to generate weights for patients on TOF to account for non-randomised treatment assignment and for whether outcomes were observed at follow-up

4. Calculate difference in weighted DAS28CRP between treatment groups at 3 and at 9 months

© 2023 Deakin CT et al. *JAMA Network Open.*

eMethods 5: Multiple Imputation

RF-MI was used to impute missing DAS28CRP components as there were many non-normal variables in the dataset which were thought to have complex, non-linear relationships. The natural order of ordinal categorical variables was maintained. Since the formula for DAS28CRP, below, uses log-transformed CRP and square root-transformed joint counts, the transformed versions of these variables were used in the imputation algorithm.

$$DAS28CRP = (0.56 * \sqrt{TJC28}) + (0.28 * \sqrt{SJC28}) + (0.36 * \log(CRP + 1)) + (0.014 * PtGA) + 0.96$$

Missing data patterns

The combination of patients with missing data at any of the timepoints is described in eTable 5, with values of 1 indicating that at least one component of DAS28CRP was recorded at that timepoint.

eTable 5. Patterns of missing DAS28CRP components at baseline and follow-up timepoints for all included patients

Baseline	3 months	9 months	Number of patients	Proportion of patients
0	0	1	45	0.05
0	1	0	25	0.03
0	1	1	38	0.05
1	0	0	143	0.17
1	0	1	124	0.15
1	1	0	169	0.20
1	1	1	298	0.35

There are 734 patients with any DAS28CRP components at 0 months (87%), regardless of missing data at follow-up. There are (108) patients with some DAS28CRP components recorded at 3 or at 9 months or at both follow-up timepoints, but who do not have any DAS28CRP components recorded at 0 months (13%).

There are 699 patients with any DAS28CRP components at 3 months or 9 months or both (83%), regardless of missing data at 0 months.

There are 143 patients with DAS28CRP components at 0 months but no subsequent DAS28CRP components (17%).

© 2023 Deakin CT et al. *JAMA Network Open.*

There are 0 patients with no DAS28CRP components at 0 months but no DAS28CRP components at 3 or 9 months (0%).

The data do not appear to be monotone missing i.e. it is not the case that if a patient's data are missing at one visit, then the data are also missing at all subsequent visits for that patient.

The combination of patients with any DAS28CRP components at 3 or at 9 months follow-up for those with baseline DAS28CRP components observed is described in eTable 6.

eTable 6. Patterns of missing DAS28CRP components at follow-up timepoints for patients with DAS28CRP components recorded at baseline

3 months	9 months	Number of patients	Proportion of patients
0	0	143	0.19
0	1	124	0.17
1	0	169	0.23
1	1	298	0.41

There are 143 patients with data on DAS28CRP components at baseline but not at 3 or 9 months. These patients were not included in the analysis that estimated the treatment effect at these follow-up timepoints, but were included in the imputation model to contribute information about the associations between DAS28CRP components at baseline and other baseline components.

Methodology for multiple imputation

RF-MI was performed using the `mice` package [6], generating `m`=10 imputed datasets. DAS28CRP was calculated from its imputed components in the complete datasets (see code).

© 2023 Deakin CT et al. *JAMA Network Open.*

eMethods 6: Stable Balancing Weights

Methodology for stable balancing weights

SBW calculates weights for patients who receive ADA and whose outcomes are observed, $(T, S) = (0,1)$ and for patients who receive TOF and whose outcomes are observed, $(T, S) = (1,1)$. Weights are not calculated for patients whose outcomes are not observed, $S = 0$.

The weights aim to balance the empirical distributions of the observed covariates, (X, Y_0), between the treatment groups for patients whose outcomes are observed, and also to balance these distributions with those for the entire sample (patients with at least one observed Y_0, Y_1 or Y_2, n=842).

SBW was run using the `sbw` package [10] (see code). Note that the balance grid (0.002, 0.005,0.01,0.02,0.05,0.1,0.25,0.4) represents the possible values considered for ‘tolerance’, a value which when multiplied by the standard deviation for each baseline characteristic produces the maximum permitted difference in means for the baseline characteristics after weighting. The algorithm selects the smallest tolerance value. The two highest tolerance values, 0.25 and 0.4, were necessary for a small number of bootstrap samples in which fewer patients in the TOF group were selected.

© 2023 Deakin CT et al. *JAMA Network Open.*

eMethods 7: Bootstrapping Procedure

To estimate a 95% confidence interval for the ATE using the percentile method, the entire procedure (i.e. steps 1-4) was repeated in $B = 1000$ bootstrap samples drawn from the original sample of n=842 patients, with replacement.

This was implemented using a series of nested for-loops, with the outer loop representing the B bootstrap samples and the inner loop representing the m imputed datasets.

The percentile method for calculating the 95% confidence interval for the point estimates was implemented (see code).

© 2023 Deakin CT et al. *JAMA Network Open.*

eResults 1: Performance of Multiple Imputation

Algorithm convergence for the imputation of the missing DAS28CRP components is shown in eFigure 5. As expected, the trace lines intermingle and there are no major trends at later iterations.

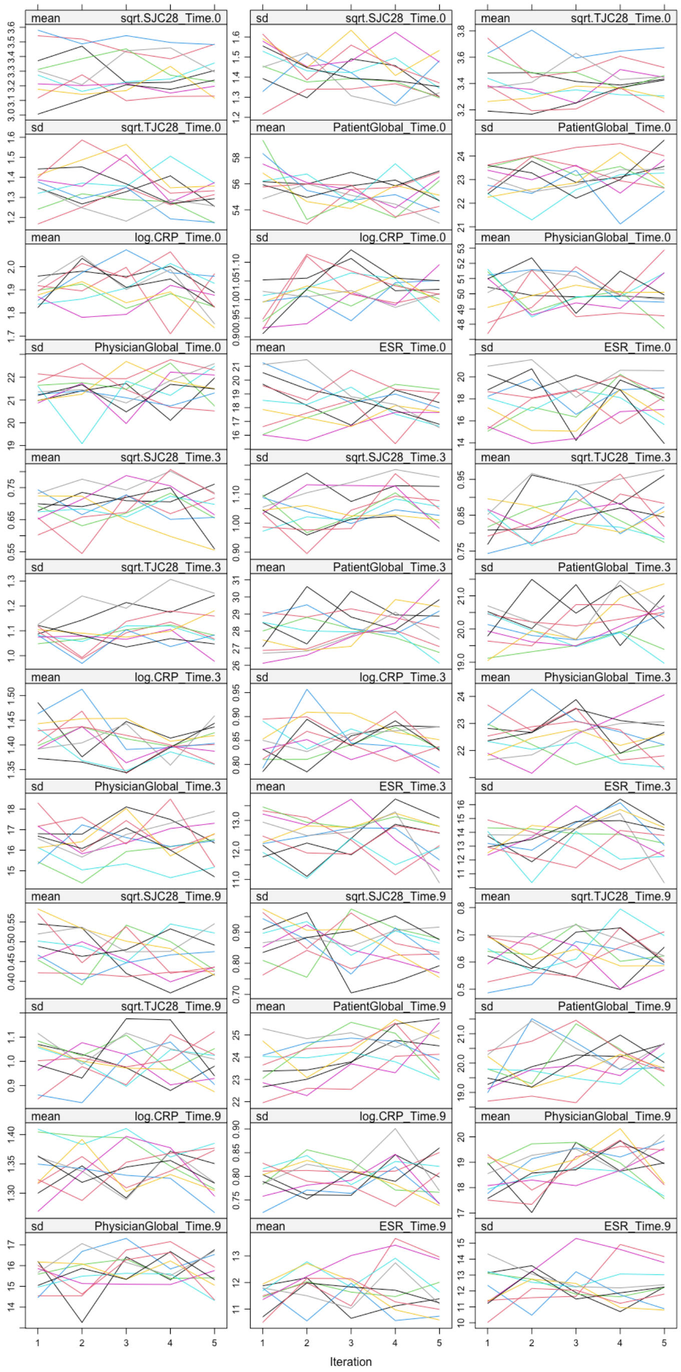

eFigure 5: Convergence of the imputation algorithm.

© 2023 Deakin CT et al. *JAMA Network Open.*

Distributions of the observed and imputed data for the transformed SJC28, TJC28 and CRP variables, and for the patient global, the physician global and ESR are depicted in eFigure 6. Although the observed data (blue) for these disease activity variables have non-normal distributions, the imputed data (red) have similar distributions indicating the RF-MI algorithm has generated plausible values.

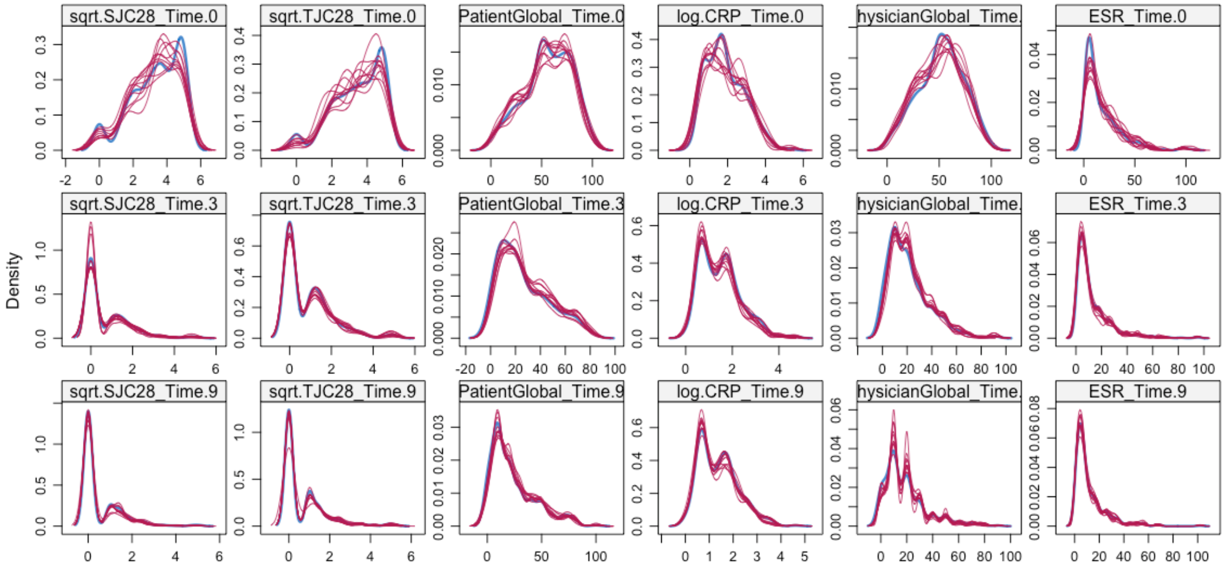

eFigure 6: Density plot to depict distributions of observed (blue) and imputed (red) data.

© 2023 Deakin CT et al. *JAMA Network Open.*

eResults 2: Properties of Stable Balancing Weights

Within each imputed dataset, the properties of weights were checked. Balance was also checked (eResults3) for the observed ADA and TOF groups, and for both groups against the whole sample of patients with observed and unobserved DAS28CRP at follow-up.

The properties of the weights estimated using SBW are described in eTable 7, with the minimum and maximum values of weights giving an indication of the dispersion of the weights. Patients who did not have any observed DAS28CRP components at follow-up had a weight of 0.

eTable 7. Properties of stable balancing weights across combinations of observed and unobserved treatment groups in each imputed dataset

Group	Imputed dataset	Mean	Minimum	Maximum	Sum
ADA, observed	1	0.002155	0.000430	0.004933	1
	2	0.002155	0.000524	0.004830	1
	3	0.002155	0.000536	0.004837	1
	4	0.002155	0.000409	0.004977	1
	5	0.002155	0.000510	0.004830	1
	6	0.002155	0.000425	0.004953	1
	7	0.002155	0.000650	0.004682	1
	8	0.002155	0.000527	0.004853	1
	9	0.002155	0.000415	0.004920	1
	10	0.002155	0.000521	0.000521	1
TOF, observed	1	0.004255	0.000000	0.017994	1
	2	0.004255	0.000000	0.018040	1
	3	0.004255	0.000000	0.017992	1
	4	0.004255	0.000000	0.018018	1
	5	0.004255	0.000000	0.018009	1
	6	0.004255	0.000000	0.017982	1
	7	0.004255	0.000000	0.017989	1
	8	0.004255	0.000000	0.018078	1

© 2023 Deakin CT et al. *JAMA Network Open.*

Group	Imputed dataset	Mean	Minimum	Maximum	Sum
	9	0.004255	0.000000	0.018053	1
	10	0.004255	0.004255	0.004255	1
All, observed	1	0.002861	0.000000	0.017994	2
	2	0.002861	0.000000	0.018040	2
	3	0.002861	0.000000	0.017992	2
	4	0.002861	0.000000	0.018018	2
	5	0.002861	0.000000	0.018009	2
	6	0.002861	0.000000	0.017982	2
	7	0.002861	0.000000	0.017989	2
	8	0.002861	0.000000	0.018078	2
	9	0.002861	0.000000	0.018053	2
	10	0.002861	0.000000	0.017906	2

© 2023 Deakin CT et al. *JAMA Network Open.*

eResults 3: Balance Checks

Balance in the baseline characteristics between treatment groups and against the entire sample (patients with at least one observed Y_0, Y_1 or Y_2, n=842) was checked within each of the m imputed datasets.

Within each imputed dataset, the baseline covariates including baseline DAS28CRP were multiplied by the weights for each individual. The mean for these variables in each treatment group are summarised below, along with the mean and standard deviation for each variable in the entire sample without weighting. The standardised mean difference for each covariate shown in eFigure 3 is the difference between treatment groups in the mean for that covariate divided by the standard deviation for the entire sample.

The maximum permitted differences in means for each covariate are also summarised. These values are equal to the standard deviation multiplied by a tolerance value for each treatment group. In a perfectly balanced dataset, the maximum differences in means for each covariate would be zero.

After applying the SBW, the maximum difference between the mean of each baseline characteristic in the ADA and TOF groups was less than 0.03% of the corresponding standard deviation in the entire sample, indicating reasonable balance was achieved.

© 2023 Deakin CT et al. *JAMA Network Open.*

Imputed dataset 1

The tolerance value for ADA was 0.002 and the tolerance value for TOF was 0.02.

eTable 8. Weighted covariate means for observed patients in treatment groups and unweighted covariate means and standard deviations for the entire sample (Imputed dataset 1)

Characteristic	Weighted mean (ADA)	Weighted mean (TOF)	Unweighted mean (entire sample)	Unweighted standard deviation (entire sample)	Maximum permitted difference between means (ADA)	Maximum permitted difference between means (TOF)
Age	56.2834	56.5756	56.3100	13.2794	0.0266	0.2656
Gender_Male	0.2924	0.2842	0.2933	0.4556	0.0009	0.0091
State_NSW	0.3043	0.3144	0.3052	0.4608	0.0009	0.0092
State_QLD	0.1336	0.1410	0.1342	0.3411	0.0007	0.0068
State_ACT	0.0790	0.0850	0.0796	0.2708	0.0005	0.0054
State_TAS	0.0494	0.0542	0.0499	0.2178	0.0004	0.0044
PatientRegional_InnerRegional	0.3822	0.3910	0.3812	0.4860	0.0010	0.0097
PatientRegional_OuterRegionalRemote	0.1027	0.1094	0.1033	0.3046	0.0006	0.0061
Duration	1.4030	1.4239	1.4049	0.9511	0.0019	0.0190
YearStart_2017	0.8889	0.8907	0.8895	0.3136	0.0006	0.0063
YearStart_2018	0.6867	0.6784	0.6876	0.4637	0.0009	0.0093
YearStart_2019	0.4784	0.4674	0.4774	0.4998	0.0010	0.0100

© 2023 Deakin CT et al. *JAMA Network Open.*

Characteristic	Weighted mean (ADA)	Weighted mean (TOF)	Unweighted mean (entire sample)	Unweighted standard deviation (entire sample)	Maximum permitted difference between means (ADA)	Maximum permitted difference between means (TOF)
YearStart_2020	0.2610	0.2513	0.2601	0.4389	0.0009	0.0088
YearStart_2021	0.0459	0.0421	0.0463	0.2103	0.0004	0.0042
NurseOnSite	0.4262	0.4153	0.4252	0.4947	0.0010	0.0099
PractitionerGender_Female	0.4111	0.4023	0.4121	0.4925	0.0010	0.0099
PractitionerExperience_16_30y	0.6891	0.6993	0.6900	0.4628	0.0009	0.0093
PractitionerExperience_over30y	0.2538	0.2443	0.2530	0.4350	0.0009	0.0087
PractitionerPtGlobal_76_100	0.5912	0.5804	0.5903	0.4921	0.0010	0.0098
BiologicUse_26_100	0.1560	0.1640	0.1568	0.3638	0.0007	0.0073
PriorRenal	0.4254	0.4363	0.4264	0.4948	0.0010	0.0099
PriorMTX	0.6411	0.6467	0.6401	0.4802	0.0010	0.0096
PriorHCQ	0.3957	0.4065	0.3967	0.4895	0.0010	0.0098
PriorLFN	0.3031	0.3132	0.3040	0.4603	0.0009	0.0092
PriorSFZ	0.2764	0.2813	0.2755	0.4470	0.0009	0.0089
PriorOCS	0.5746	0.5641	0.5736	0.4948	0.0010	0.0099
ConcomitantMTX	0.2723	0.2673	0.2732	0.4458	0.0009	0.0089
ConcomitantHCQ	0.2408	0.2314	0.2399	0.4273	0.0009	0.0085
ConcomitantLFN	0.1789	0.1817	0.1781	0.3829	0.0008	0.0077

© 2023 Deakin CT et al. *JAMA Network Open.*

Characteristic	Weighted mean (ADA)	Weighted mean (TOF)	Unweighted mean (entire sample)	Unweighted standard deviation (entire sample)	Maximum permitted difference between means (ADA)	Maximum permitted difference between means (TOF)
ConcomitantSFZ	0.1513	0.1592	0.1520	0.3593	0.0007	0.0072
ConcomitantOCS	0.4915	0.4805	0.4905	0.5002	0.0010	0.0100
DAS28CRP_Time.0	5.3070	5.2838	5.3095	1.2859	0.0026	0.0257

Imputed dataset 2

The tolerance value for ADA was 0.005 and the tolerance value for TOF was 0.02.

eTable 9. Weighted covariate means for observed patients in treatment groups and unweighted covariate means and standard deviations for the entire sample (Imputed dataset 2)

Characteristic	Weighted mean (ADA)	Weighted mean (TOF)	Unweighted mean (entire sample)	Unweighted standard deviation (entire sample)	Maximum permitted difference between means (ADA)	Maximum permitted difference between means (TOF)
Age	56.2436	56.5756	56.3100	13.2794	0.0664	0.2656
Gender_Male	0.2911	0.2842	0.2933	0.4556	0.0023	0.0091
State_NSW	0.3029	0.3144	0.3052	0.4608	0.0023	0.0092
State_QLD	0.1337	0.1410	0.1342	0.3411	0.0017	0.0068
State_ACT	0.0782	0.0850	0.0796	0.2708	0.0014	0.0054

© 2023 Deakin CT et al. *JAMA Network Open.*

Characteristic	Weighted mean (ADA)	Weighted mean (TOF)	Unweighted mean (entire sample)	Unweighted standard deviation (entire sample)	Maximum permitted difference between means (ADA)	Maximum permitted difference between means (TOF)
State_TAS	0.0488	0.0542	0.0499	0.2178	0.0011	0.0044
PatientRegional_InnerRegional	0.3821	0.3910	0.3812	0.4860	0.0024	0.0097
PatientRegional_OuterRegionalRemote	0.1018	0.1094	0.1033	0.3046	0.0015	0.0061
Duration	1.4002	1.4239	1.4049	0.9511	0.0048	0.0190
YearStart_2017	0.8880	0.8894	0.8895	0.3136	0.0016	0.0063
YearStart_2018	0.6853	0.6784	0.6876	0.4637	0.0023	0.0093
YearStart_2019	0.4799	0.4674	0.4774	0.4998	0.0025	0.0100
YearStart_2020	0.2623	0.2513	0.2601	0.4389	0.0022	0.0088
YearStart_2021	0.0453	0.0421	0.0463	0.2103	0.0011	0.0042
NurseOnSite	0.4277	0.4153	0.4252	0.4947	0.0025	0.0099
PractitionerGender_Female	0.4097	0.4023	0.4121	0.4925	0.0025	0.0099
PractitionerExperience_16_30y	0.6877	0.6993	0.6900	0.4628	0.0023	0.0093
PractitionerExperience_over30y	0.2551	0.2443	0.2530	0.4350	0.0022	0.0087
PractitionerPtGlobal_76_100	0.5927	0.5804	0.5903	0.4921	0.0025	0.0098
BiologicUse_26_100	0.1550	0.1640	0.1568	0.3638	0.0018	0.0073
PriorRenal	0.4239	0.4363	0.4264	0.4948	0.0025	0.0099
PriorMTX	0.6425	0.6465	0.6401	0.4802	0.0024	0.0096

© 2023 Deakin CT et al. *JAMA Network Open.*

Characteristic	Weighted mean (ADA)	Weighted mean (TOF)	Unweighted mean (entire sample)	Unweighted standard deviation (entire sample)	Maximum permitted difference between means (ADA)	Maximum permitted difference between means (TOF)
PriorHCQ	0.3942	0.4065	0.3967	0.4895	0.0024	0.0098
PriorLFN	0.3017	0.3132	0.3040	0.4603	0.0023	0.0092
PriorSFZ	0.2778	0.2811	0.2755	0.4470	0.0022	0.0089
PriorOCS	0.5761	0.5639	0.5736	0.4948	0.0025	0.0099
ConcomitantMTX	0.2719	0.2665	0.2732	0.4458	0.0022	0.0089
ConcomitantHCQ	0.2420	0.2314	0.2399	0.4273	0.0021	0.0085
ConcomitantLFN	0.1801	0.1822	0.1781	0.3829	0.0019	0.0077
ConcomitantSFZ	0.1502	0.1592	0.1520	0.3593	0.0018	0.0072
ConcomitantOCS	0.4930	0.4805	0.4905	0.5002	0.0025	0.0100
DAS28CRP_Time.0	5.3323	5.3004	5.3259	1.2735	0.0064	0.0255

Imputed dataset 3

The tolerance value for ADA was 0.005 and the tolerance value for TOF was 0.02.

© 2023 Deakin CT et al. *JAMA Network Open.*

eTable 10. Weighted covariate means for observed patients in treatment groups and unweighted covariate means and standard deviations for the entire sample (Imputed dataset 3)

Characteristic	Weighted mean (ADA)	Weighted mean (TOF)	Unweighted mean (entire sample)	Unweighted standard deviation (entire sample)	Maximum permitted difference between means (ADA)	Maximum permitted difference between means (TOF)
Age	56.2436	56.5756	56.3100	13.2794	0.0664	0.2656
Gender_Male	0.2912	0.2842	0.2933	0.4556	0.0023	0.0091
State_NSW	0.3029	0.3144	0.3052	0.4608	0.0023	0.0092
State_QLD	0.1337	0.1410	0.1342	0.3411	0.0017	0.0068
State_ACT	0.0782	0.0850	0.0796	0.2708	0.0014	0.0054
State_TAS	0.0488	0.0542	0.0499	0.2178	0.0011	0.0044
PatientRegional_InnerRegional	0.3822	0.3910	0.3812	0.4860	0.0024	0.0097
PatientRegional_OuterRegionalRemote	0.1018	0.1094	0.1033	0.3046	0.0015	0.0061
Duration	1.4002	1.4239	1.4049	0.9511	0.0048	0.0190
YearStart_2017	0.8880	0.8897	0.8895	0.3136	0.0016	0.0063
YearStart_2018	0.6853	0.6784	0.6876	0.4637	0.0023	0.0093
YearStart_2019	0.4799	0.4674	0.4774	0.4998	0.0025	0.0100
YearStart_2020	0.2623	0.2513	0.2601	0.4389	0.0022	0.0088
YearStart_2021	0.0453	0.0421	0.0463	0.2103	0.0011	0.0042
NurseOnSite	0.4277	0.4153	0.4252	0.4947	0.0025	0.0099

© 2023 Deakin CT et al. *JAMA Network Open.*

Characteristic	Weighted mean (ADA)	Weighted mean (TOF)	Unweighted mean (entire sample)	Unweighted standard deviation (entire sample)	Maximum permitted difference between means (ADA)	Maximum permitted difference between means (TOF)
PractitionerGender_Female	0.4097	0.4023	0.4121	0.4925	0.0025	0.0099
PractitionerExperience_16_30y	0.6877	0.6993	0.6900	0.4628	0.0023	0.0093
PractitionerExperience_over30y	0.2551	0.2443	0.2530	0.4350	0.0022	0.0087
PractitionerPtGlobal_76_100	0.5927	0.5804	0.5903	0.4921	0.0025	0.0098
BiologicUse_26_100	0.1550	0.1640	0.1568	0.3638	0.0018	0.0073
PriorRenal	0.4239	0.4363	0.4264	0.4948	0.0025	0.0099
PriorMTX	0.6425	0.6473	0.6401	0.4802	0.0024	0.0096
PriorHCQ	0.3942	0.4065	0.3967	0.4895	0.0024	0.0098
PriorLFN	0.3017	0.3132	0.3040	0.4603	0.0023	0.0092
PriorSFZ	0.2778	0.2820	0.2755	0.4470	0.0022	0.0089
PriorOCS	0.5761	0.5652	0.5736	0.4948	0.0025	0.0099
ConcomitantMTX	0.2723	0.2668	0.2732	0.4458	0.0022	0.0089
ConcomitantHCQ	0.2420	0.2314	0.2399	0.4273	0.0021	0.0085
ConcomitantLFN	0.1801	0.1816	0.1781	0.3829	0.0019	0.0077
ConcomitantSFZ	0.1502	0.1592	0.1520	0.3593	0.0018	0.0072
ConcomitantOCS	0.4930	0.4805	0.4905	0.5002	0.0025	0.0100
DAS28CRP_Time.0	5.3079	5.2759	5.3015	1.2809	0.0064	0.0256

© 2023 Deakin CT et al. *JAMA Network Open.*

Imputed dataset 4

The tolerance value for ADA was 0.002 and the tolerance value for TOF was 0.02.

eTable 11. Weighted covariate means for observed patients in treatment groups and unweighted covariate means and standard deviations for the entire sample (Imputed dataset 4)

Characteristic	Weighted mean (ADA)	Weighted mean (TOF)	Unweighted mean (entire sample)	Unweighted standard deviation (entire sample)	Maximum permitted difference between means (ADA)	Maximum permitted difference between means (TOF)
Age	56.2834	56.5756	56.3100	13.2794	0.0266	0.2656
Gender_Male	0.2924	0.2842	0.2933	0.4556	0.0009	0.0091
State_NSW	0.3043	0.3144	0.3052	0.4608	0.0009	0.0092
State_QLD	0.1338	0.1410	0.1342	0.3411	0.0007	0.0068
State_ACT	0.0790	0.0850	0.0796	0.2708	0.0005	0.0054
State_TAS	0.0494	0.0542	0.0499	0.2178	0.0004	0.0044
PatientRegional_InnerRegional	0.3822	0.3910	0.3812	0.4860	0.0010	0.0097
PatientRegional_OuterRegionalRemote	0.1027	0.1094	0.1033	0.3046	0.0006	0.0061
Duration	1.4030	1.4239	1.4049	0.9511	0.0019	0.0190
YearStart_2017	0.8889	0.8899	0.8895	0.3136	0.0006	0.0063
YearStart_2018	0.6867	0.6784	0.6876	0.4637	0.0009	0.0093
YearStart_2019	0.4784	0.4674	0.4774	0.4998	0.0010	0.0100

Characteristic	Weighted mean (ADA)	Weighted mean (TOF)	Unweighted mean (entire sample)	Unweighted standard deviation (entire sample)	Maximum permitted difference between means (ADA)	Maximum permitted difference between means (TOF)
YearStart_2020	0.2610	0.2513	0.2601	0.4389	0.0009	0.0088
YearStart_2021	0.0459	0.0421	0.0463	0.2103	0.0004	0.0042
NurseOnSite	0.4262	0.4153	0.4252	0.4947	0.0010	0.0099
PractitionerGender_Female	0.4111	0.4023	0.4121	0.4925	0.0010	0.0099
PractitionerExperience_16_30y	0.6891	0.6993	0.6900	0.4628	0.0009	0.0093
PractitionerExperience_over30y	0.2538	0.2443	0.2530	0.4350	0.0009	0.0087
PractitionerPtGlobal_76_100	0.5912	0.5804	0.5903	0.4921	0.0010	0.0098
BiologicUse_26_100	0.1560	0.1640	0.1568	0.3638	0.0007	0.0073
PriorRenal	0.4254	0.4363	0.4264	0.4948	0.0010	0.0099
PriorMTX	0.6411	0.6468	0.6401	0.4802	0.0010	0.0096
PriorHCQ	0.3957	0.4065	0.3967	0.4895	0.0010	0.0098
PriorLFN	0.3031	0.3132	0.3040	0.4603	0.0009	0.0092
PriorSFZ	0.2764	0.2815	0.2755	0.4470	0.0009	0.0089
PriorOCS	0.5746	0.5644	0.5736	0.4948	0.0010	0.0099
ConcomitantMTX	0.2723	0.2676	0.2732	0.4458	0.0009	0.0089
ConcomitantHCQ	0.2408	0.2314	0.2399	0.4273	0.0009	0.0085
ConcomitantLFN	0.1789	0.1815	0.1781	0.3829	0.0008	0.0077

© 2023 Deakin CT et al. *JAMA Network Open.*

Characteristic	Weighted mean (ADA)	Weighted mean (TOF)	Unweighted mean (entire sample)	Unweighted standard deviation (entire sample)	Maximum permitted difference between means (ADA)	Maximum permitted difference between means (TOF)
ConcomitantSFZ	0.1513	0.1592	0.1520	0.3593	0.0007	0.0072
ConcomitantOCS	0.4915	0.4805	0.4905	0.5002	0.0010	0.0100
DAS28CRP_Time.0	5.3218	5.2935	5.3192	1.2854	0.0026	0.0257

Imputed dataset 5

The tolerance value for ADA was 0.005 and the tolerance value for TOF was 0.02.

eTable 12. Weighted covariate means for observed patients in treatment groups and unweighted covariate means and standard deviations for the entire sample (Imputed dataset 5)

Characteristic	Weighted mean (ADA)	Weighted mean (TOF)	Unweighted mean (entire sample)	Unweighted standard deviation (entire sample)	Maximum permitted difference between means (ADA)	Maximum permitted difference between means (TOF)
Age	56.2436	56.5756	56.3100	13.2794	0.0664	0.2656
Gender_Male	0.2912	0.2842	0.2933	0.4556	0.0023	0.0091
State_NSW	0.3029	0.3144	0.3052	0.4608	0.0023	0.0092
State_QLD	0.1337	0.1410	0.1342	0.3411	0.0017	0.0068
State_ACT	0.0782	0.0850	0.0796	0.2708	0.0014	0.0054

© 2023 Deakin CT et al. *JAMA Network Open.*

Characteristic	Weighted mean (ADA)	Weighted mean (TOF)	Unweighted mean (entire sample)	Unweighted standard deviation (entire sample)	Maximum permitted difference between means (ADA)	Maximum permitted difference between means (TOF)
State_TAS	0.0488	0.0542	0.0499	0.2178	0.0011	0.0044
PatientRegional_InnerRegional	0.3823	0.3910	0.3812	0.4860	0.0024	0.0097
PatientRegional_OuterRegionalRemote	0.1018	0.1094	0.1033	0.3046	0.0015	0.0061
Duration	1.4002	1.4239	1.4049	0.9511	0.0048	0.0190
YearStart_2017	0.8880	0.8920	0.8895	0.3136	0.0016	0.0063
YearStart_2018	0.6853	0.6784	0.6876	0.4637	0.0023	0.0093
YearStart_2019	0.4799	0.4674	0.4774	0.4998	0.0025	0.0100
YearStart_2020	0.2623	0.2513	0.2601	0.4389	0.0022	0.0088
YearStart_2021	0.0453	0.0421	0.0463	0.2103	0.0011	0.0042
NurseOnSite	0.4277	0.4153	0.4252	0.4947	0.0025	0.0099
PractitionerGender_Female	0.4097	0.4023	0.4121	0.4925	0.0025	0.0099
PractitionerExperience_16_30y	0.6877	0.6993	0.6900	0.4628	0.0023	0.0093
PractitionerExperience_over30y	0.2551	0.2443	0.2530	0.4350	0.0022	0.0087
PractitionerPtGlobal_76_100	0.5927	0.5804	0.5903	0.4921	0.0025	0.0098
BiologicUse_26_100	0.1550	0.1640	0.1568	0.3638	0.0018	0.0073
PriorRenal	0.4239	0.4363	0.4264	0.4948	0.0025	0.0099
PriorMTX	0.6425	0.6472	0.6401	0.4802	0.0024	0.0096

© 2023 Deakin CT et al. *JAMA Network Open.*

Characteristic	Weighted mean (ADA)	Weighted mean (TOF)	Unweighted mean (entire sample)	Unweighted standard deviation (entire sample)	Maximum permitted difference between means (ADA)	Maximum permitted difference between means (TOF)
PriorHCQ	0.3942	0.4065	0.3967	0.4895	0.0024	0.0098
PriorLFN	0.3017	0.3132	0.3040	0.4603	0.0023	0.0092
PriorSFZ	0.2778	0.2840	0.2755	0.4470	0.0022	0.0089
PriorOCS	0.5761	0.5653	0.5736	0.4948	0.0025	0.0099
ConcomitantMTX	0.2721	0.2659	0.2732	0.4458	0.0022	0.0089
ConcomitantHCQ	0.2420	0.2314	0.2399	0.4273	0.0021	0.0085
ConcomitantLFN	0.1801	0.1805	0.1781	0.3829	0.0019	0.0077
ConcomitantSFZ	0.1502	0.1592	0.1520	0.3593	0.0018	0.0072
ConcomitantOCS	0.4930	0.4805	0.4905	0.5002	0.0025	0.0100
DAS28CRP_Time.0	5.2982	5.2655	5.2916	1.3075	0.0065	0.0262

Imputed dataset 6

The tolerance value for ADA was 0.002 and the tolerance value for TOF was 0.02.

© 2023 Deakin CT et al. *JAMA Network Open.*

eTable 13. Weighted covariate means for observed patients in treatment groups and unweighted covariate means and standard deviations for the entire sample (Imputed dataset 6)

Characteristic	Weighted mean (ADA)	Weighted mean (TOF)	Unweighted mean (entire sample)	Unweighted standard deviation (entire sample)	Maximum permitted difference between means (ADA)	Maximum permitted difference between means (TOF)
Age	56.2834	56.5756	56.3100	13.2794	0.0266	0.2656
Gender_Male	0.2924	0.2842	0.2933	0.4556	0.0009	0.0091
State_NSW	0.3043	0.3144	0.3052	0.4608	0.0009	0.0092
State_QLD	0.1337	0.1410	0.1342	0.3411	0.0007	0.0068
State_ACT	0.0790	0.0850	0.0796	0.2708	0.0005	0.0054
State_TAS	0.0494	0.0542	0.0499	0.2178	0.0004	0.0044
PatientRegional_InnerRegional	0.3822	0.3910	0.3812	0.4860	0.0010	0.0097
PatientRegional_OuterRegionalRemote	0.1027	0.1094	0.1033	0.3046	0.0006	0.0061
Duration	1.4030	1.4239	1.4049	0.9511	0.0019	0.0190
YearStart_2017	0.8889	0.8898	0.8895	0.3136	0.0006	0.0063
YearStart_2018	0.6867	0.6784	0.6876	0.4637	0.0009	0.0093
YearStart_2019	0.4784	0.4674	0.4774	0.4998	0.0010	0.0100
YearStart_2020	0.2610	0.2513	0.2601	0.4389	0.0009	0.0088
YearStart_2021	0.0459	0.0421	0.0463	0.2103	0.0004	0.0042
NurseOnSite	0.4262	0.4153	0.4252	0.4947	0.0010	0.0099

© 2023 Deakin CT et al. *JAMA Network Open.*

Characteristic	Weighted mean (ADA)	Weighted mean (TOF)	Unweighted mean (entire sample)	Unweighted standard deviation (entire sample)	Maximum permitted difference between means (ADA)	Maximum permitted difference between means (TOF)
PractitionerGender_Female	0.4111	0.4023	0.4121	0.4925	0.0010	0.0099
PractitionerExperience_16_30y	0.6891	0.6993	0.6900	0.4628	0.0009	0.0093
PractitionerExperience_over30y	0.2538	0.2443	0.2530	0.4350	0.0009	0.0087
PractitionerPtGlobal_76_100	0.5912	0.5804	0.5903	0.4921	0.0010	0.0098
BiologicUse_26_100	0.1560	0.1640	0.1568	0.3638	0.0007	0.0073
PriorRenal	0.4254	0.4363	0.4264	0.4948	0.0010	0.0099
PriorMTX	0.6411	0.6477	0.6401	0.4802	0.0010	0.0096
PriorHCQ	0.3957	0.4065	0.3967	0.4895	0.0010	0.0098
PriorLFN	0.3031	0.3132	0.3040	0.4603	0.0009	0.0092
PriorSFZ	0.2764	0.2818	0.2755	0.4470	0.0009	0.0089
PriorOCS	0.5746	0.5650	0.5736	0.4948	0.0010	0.0099
ConcomitantMTX	0.2723	0.2672	0.2732	0.4458	0.0009	0.0089
ConcomitantHCQ	0.2408	0.2314	0.2399	0.4273	0.0009	0.0085
ConcomitantLFN	0.1789	0.1820	0.1781	0.3829	0.0008	0.0077
ConcomitantSFZ	0.1513	0.1592	0.1520	0.3593	0.0007	0.0072
ConcomitantOCS	0.4915	0.4805	0.4905	0.5002	0.0010	0.0100
DAS28CRP_Time.0	5.3062	5.2776	5.3036	1.3008	0.0026	0.0260

© 2023 Deakin CT et al. *JAMA Network Open.*

Imputed dataset 7

The tolerance value for ADA was 0.01 and the tolerance value for TOF was 0.02.

eTable 14. Weighted covariate means for observed patients in treatment groups and unweighted covariate means and standard deviations for the entire sample (Imputed dataset 7)

Characteristic	Weighted mean (ADA)	Weighted mean (TOF)	Unweighted mean (entire sample)	Unweighted standard deviation (entire sample)	Maximum permitted difference between means (ADA)	Maximum permitted difference between means (TOF)
Age	56.1772	56.5756	56.3100	13.2794	0.1328	0.2656
Gender_Male	0.2922	0.2842	0.2933	0.4556	0.0046	0.0091
State_NSW	0.3006	0.3144	0.3052	0.4608	0.0046	0.0092
State_QLD	0.1337	0.1410	0.1342	0.3411	0.0034	0.0068
State_ACT	0.0769	0.0850	0.0796	0.2708	0.0027	0.0054
State_TAS	0.0477	0.0542	0.0499	0.2178	0.0022	0.0044
PatientRegional_InnerRegional	0.3812	0.3910	0.3812	0.4860	0.0049	0.0097
PatientRegional_OuterRegionalRemote	0.1003	0.1094	0.1033	0.3046	0.0030	0.0061
Duration	1.3954	1.4239	1.4049	0.9511	0.0095	0.0190
YearStart_2017	0.8864	0.8900	0.8895	0.3136	0.0031	0.0063
YearStart_2018	0.6830	0.6784	0.6876	0.4637	0.0046	0.0093
YearStart_2019	0.4824	0.4674	0.4774	0.4998	0.0050	0.0100

© 2023 Deakin CT et al. *JAMA Network Open.*

Characteristic	Weighted mean (ADA)	Weighted mean (TOF)	Unweighted mean (entire sample)	Unweighted standard deviation (entire sample)	Maximum permitted difference between means (ADA)	Maximum permitted difference between means (TOF)
YearStart_2020	0.2645	0.2513	0.2601	0.4389	0.0044	0.0088
YearStart_2021	0.0442	0.0421	0.0463	0.2103	0.0021	0.0042
NurseOnSite	0.4301	0.4153	0.4252	0.4947	0.0049	0.0099
PractitionerGender_Female	0.4072	0.4023	0.4121	0.4925	0.0049	0.0099
PractitionerExperience_16_30y	0.6854	0.6993	0.6900	0.4628	0.0046	0.0093
PractitionerExperience_over30y	0.2573	0.2443	0.2530	0.4350	0.0043	0.0087
PractitionerPtGlobal_76_100	0.5952	0.5804	0.5903	0.4921	0.0049	0.0098
BiologicUse_26_100	0.1531	0.1640	0.1568	0.3638	0.0036	0.0073
PriorRenal	0.4227	0.4363	0.4264	0.4948	0.0049	0.0099
PriorMTX	0.6449	0.6473	0.6401	0.4802	0.0048	0.0096
PriorHCQ	0.3918	0.4065	0.3967	0.4895	0.0049	0.0098
PriorLFN	0.2994	0.3132	0.3040	0.4603	0.0046	0.0092
PriorSFZ	0.2800	0.2814	0.2755	0.4470	0.0045	0.0089
PriorOCS	0.5786	0.5645	0.5736	0.4948	0.0049	0.0099
ConcomitantMTX	0.2735	0.2677	0.2732	0.4458	0.0045	0.0089
ConcomitantHCQ	0.2442	0.2314	0.2399	0.4273	0.0043	0.0085
ConcomitantLFN	0.1820	0.1814	0.1781	0.3829	0.0038	0.0077

© 2023 Deakin CT et al. *JAMA Network Open.*

Characteristic	Weighted mean (ADA)	Weighted mean (TOF)	Unweighted mean (entire sample)	Unweighted standard deviation (entire sample)	Maximum permitted difference between means (ADA)	Maximum permitted difference between means (TOF)
ConcomitantSFZ	0.1518	0.1592	0.1520	0.3593	0.0036	0.0072
ConcomitantOCS	0.4955	0.4805	0.4905	0.5002	0.0050	0.0100
DAS28CRP_Time.0	5.2729	5.2484	5.2744	1.2993	0.0130	0.0260

Imputed dataset 8

The tolerance value for ADA was 0.005 and the tolerance value for TOF was 0.02.

eTable 15. Weighted covariate means for observed patients in treatment groups and unweighted covariate means and standard deviations for the entire sample (Imputed dataset 8)

Characteristic	Weighted mean (ADA)	Weighted mean (TOF)	Unweighted mean (entire sample)	Unweighted standard deviation (entire sample)	Maximum permitted difference between means (ADA)	Maximum permitted difference between means (TOF)
Age	56.2436	56.5756	56.3100	13.2794	0.0664	0.2656
Gender_Male	0.2911	0.2842	0.2933	0.4556	0.0023	0.0091
State_NSW	0.3029	0.3144	0.3052	0.4608	0.0023	0.0092
State_QLD	0.1337	0.1410	0.1342	0.3411	0.0017	0.0068
State_ACT	0.0782	0.0850	0.0796	0.2708	0.0014	0.0054

© 2023 Deakin CT et al. *JAMA Network Open.*

Characteristic	Weighted mean (ADA)	Weighted mean (TOF)	Unweighted mean (entire sample)	Unweighted standard deviation (entire sample)	Maximum permitted difference between means (ADA)	Maximum permitted difference between means (TOF)
State_TAS	0.0488	0.0542	0.0499	0.2178	0.0011	0.0044
PatientRegional_InnerRegional	0.3817	0.3910	0.3812	0.4860	0.0024	0.0097
PatientRegional_OuterRegionalRemote	0.1018	0.1094	0.1033	0.3046	0.0015	0.0061
Duration	1.4002	1.4239	1.4049	0.9511	0.0048	0.0190
YearStart_2017	0.8880	0.8902	0.8895	0.3136	0.0016	0.0063
YearStart_2018	0.6853	0.6784	0.6876	0.4637	0.0023	0.0093
YearStart_2019	0.4799	0.4674	0.4774	0.4998	0.0025	0.0100
YearStart_2020	0.2623	0.2513	0.2601	0.4389	0.0022	0.0088
YearStart_2021	0.0453	0.0421	0.0463	0.2103	0.0011	0.0042
NurseOnSite	0.4277	0.4153	0.4252	0.4947	0.0025	0.0099
PractitionerGender_Female	0.4097	0.4023	0.4121	0.4925	0.0025	0.0099
PractitionerExperience_16_30y	0.6877	0.6993	0.6900	0.4628	0.0023	0.0093
PractitionerExperience_over30y	0.2551	0.2443	0.2530	0.4350	0.0022	0.0087
PractitionerPtGlobal_76_100	0.5927	0.5804	0.5903	0.4921	0.0025	0.0098
BiologicUse_26_100	0.1550	0.1640	0.1568	0.3638	0.0018	0.0073
PriorRenal	0.4239	0.4363	0.4264	0.4948	0.0025	0.0099
PriorMTX	0.6425	0.6464	0.6401	0.4802	0.0024	0.0096

© 2023 Deakin CT et al. *JAMA Network Open.*

Characteristic	Weighted mean (ADA)	Weighted mean (TOF)	Unweighted mean (entire sample)	Unweighted standard deviation (entire sample)	Maximum permitted difference between means (ADA)	Maximum permitted difference between means (TOF)
PriorHCQ	0.3942	0.4065	0.3967	0.4895	0.0024	0.0098
PriorLFN	0.3017	0.3132	0.3040	0.4603	0.0023	0.0092
PriorSFZ	0.2778	0.2813	0.2755	0.4470	0.0022	0.0089
PriorOCS	0.5761	0.5639	0.5736	0.4948	0.0025	0.0099
ConcomitantMTX	0.2715	0.2674	0.2732	0.4458	0.0022	0.0089
ConcomitantHCQ	0.2420	0.2314	0.2399	0.4273	0.0021	0.0085
ConcomitantLFN	0.1801	0.1812	0.1781	0.3829	0.0019	0.0077
ConcomitantSFZ	0.1502	0.1592	0.1520	0.3593	0.0018	0.0072
ConcomitantOCS	0.4930	0.4805	0.4905	0.5002	0.0025	0.0100
DAS28CRP_Time.0	5.2751	5.2556	5.2816	1.3040	0.0065	0.0261

Imputed dataset 9

The tolerance value for ADA was 0.002 and the tolerance value for TOF was 0.02.

© 2023 Deakin CT et al. *JAMA Network Open.*

eTable 16. Weighted covariate means for observed patients in treatment groups and unweighted covariate means and standard deviations for the entire sample (Imputed dataset 9)

Characteristic	Weighted mean (ADA)	Weighted mean (TOF)	Unweighted mean (entire sample)	Unweighted standard deviation (entire sample)	Maximum permitted difference between means (ADA)	Maximum permitted difference between means (TOF)
Age	56.2834	56.5756	56.3100	13.2794	0.0266	0.2656
Gender_Male	0.2924	0.2842	0.2933	0.4556	0.0009	0.0091
State_NSW	0.3043	0.3144	0.3052	0.4608	0.0009	0.0092
State_QLD	0.1335	0.1410	0.1342	0.3411	0.0007	0.0068
State_ACT	0.0790	0.0850	0.0796	0.2708	0.0005	0.0054
State_TAS	0.0494	0.0542	0.0499	0.2178	0.0004	0.0044
PatientRegional_InnerRegional	0.3821	0.3910	0.3812	0.4860	0.0010	0.0097
PatientRegional_OuterRegionalRemote	0.1027	0.1094	0.1033	0.3046	0.0006	0.0061
Duration	1.4030	1.4239	1.4049	0.9511	0.0019	0.0190
YearStart_2017	0.8889	0.8895	0.8895	0.3136	0.0006	0.0063
YearStart_2018	0.6867	0.6784	0.6876	0.4637	0.0009	0.0093
YearStart_2019	0.4784	0.4674	0.4774	0.4998	0.0010	0.0100
YearStart_2020	0.2610	0.2513	0.2601	0.4389	0.0009	0.0088
YearStart_2021	0.0459	0.0421	0.0463	0.2103	0.0004	0.0042
NurseOnSite	0.4262	0.4153	0.4252	0.4947	0.0010	0.0099

© 2023 Deakin CT et al. *JAMA Network Open.*

Characteristic	Weighted mean (ADA)	Weighted mean (TOF)	Unweighted mean (entire sample)	Unweighted standard deviation (entire sample)	Maximum permitted difference between means (ADA)	Maximum permitted difference between means (TOF)
PractitionerGender_Female	0.4111	0.4023	0.4121	0.4925	0.0010	0.0099
PractitionerExperience_16_30y	0.6891	0.6993	0.6900	0.4628	0.0009	0.0093
PractitionerExperience_over30y	0.2538	0.2443	0.2530	0.4350	0.0009	0.0087
PractitionerPtGlobal_76_100	0.5912	0.5804	0.5903	0.4921	0.0010	0.0098
BiologicUse_26_100	0.1560	0.1640	0.1568	0.3638	0.0007	0.0073
PriorRenal	0.4254	0.4363	0.4264	0.4948	0.0010	0.0099
PriorMTX	0.6411	0.6467	0.6401	0.4802	0.0010	0.0096
PriorHCQ	0.3957	0.4065	0.3967	0.4895	0.0010	0.0098
PriorLFN	0.3031	0.3132	0.3040	0.4603	0.0009	0.0092
PriorSFZ	0.2764	0.2812	0.2755	0.4470	0.0009	0.0089
PriorOCS	0.5746	0.5641	0.5736	0.4948	0.0010	0.0099
ConcomitantMTX	0.2723	0.2670	0.2732	0.4458	0.0009	0.0089
ConcomitantHCQ	0.2408	0.2314	0.2399	0.4273	0.0009	0.0085
ConcomitantLFN	0.1789	0.1824	0.1781	0.3829	0.0008	0.0077
ConcomitantSFZ	0.1513	0.1592	0.1520	0.3593	0.0007	0.0072
ConcomitantOCS	0.4915	0.4805	0.4905	0.5002	0.0010	0.0100
DAS28CRP_Time.0	5.2885	5.2651	5.2911	1.2981	0.0026	0.0260

© 2023 Deakin CT et al. *JAMA Network Open.*

Imputed dataset 10

The tolerance value for ADA was 0.005 and the tolerance value for TOF was 0.02.

eTable 17. Weighted covariate means for observed patients in treatment groups and unweighted covariate means and standard deviations for the entire sample (Imputed dataset 10)

Characteristic	Weighted mean (ADA)	Weighted mean (TOF)	Unweighted mean (entire sample)	Unweighted standard deviation (entire sample)	Maximum permitted difference between means (ADA)	Maximum permitted difference between means (TOF)
Age	56.2436	56.5756	56.3100	13.2794	0.0664	0.2656
Gender_Male	0.2911	0.2842	0.2933	0.4556	0.0023	0.0091
State_NSW	0.3029	0.3144	0.3052	0.4608	0.0023	0.0092
State_QLD	0.1337	0.1410	0.1342	0.3411	0.0017	0.0068
State_ACT	0.0782	0.0850	0.0796	0.2708	0.0014	0.0054
State_TAS	0.0488	0.0542	0.0499	0.2178	0.0011	0.0044
PatientRegional_InnerRegional	0.3820	0.3910	0.3812	0.4860	0.0024	0.0097
PatientRegional_OuterRegionalRemote	0.1018	0.1094	0.1033	0.3046	0.0015	0.0061
Duration	1.4002	1.4239	1.4049	0.9511	0.0048	0.0190
YearStart_2017	0.8880	0.8909	0.8895	0.3136	0.0016	0.0063
YearStart_2018	0.6853	0.6784	0.6876	0.4637	0.0023	0.0093
YearStart_2019	0.4799	0.4674	0.4774	0.4998	0.0025	0.0100

© 2023 Deakin CT et al. *JAMA Network Open.*

Characteristic	Weighted mean (ADA)	Weighted mean (TOF)	Unweighted mean (entire sample)	Unweighted standard deviation (entire sample)	Maximum permitted difference between means (ADA)	Maximum permitted difference between means (TOF)
YearStart_2020	0.2623	0.2513	0.2601	0.4389	0.0022	0.0088
YearStart_2021	0.0453	0.0421	0.0463	0.2103	0.0011	0.0042
NurseOnSite	0.4277	0.4153	0.4252	0.4947	0.0025	0.0099
PractitionerGender_Female	0.4097	0.4023	0.4121	0.4925	0.0025	0.0099
PractitionerExperience_16_30y	0.6877	0.6993	0.6900	0.4628	0.0023	0.0093
PractitionerExperience_over30y	0.2551	0.2443	0.2530	0.4350	0.0022	0.0087
PractitionerPtGlobal_76_100	0.5927	0.5804	0.5903	0.4921	0.0025	0.0098
BiologicUse_26_100	0.1550	0.1640	0.1568	0.3638	0.0018	0.0073
PriorRenal	0.4239	0.4363	0.4264	0.4948	0.0025	0.0099
PriorMTX	0.6425	0.6484	0.6401	0.4802	0.0024	0.0096
PriorHCQ	0.3942	0.4065	0.3967	0.4895	0.0024	0.0098
PriorLFN	0.3017	0.3132	0.3040	0.4603	0.0023	0.0092
PriorSFZ	0.2778	0.2818	0.2755	0.4470	0.0022	0.0089
PriorOCS	0.5761	0.5657	0.5736	0.4948	0.0025	0.0099
ConcomitantMTX	0.2718	0.2679	0.2732	0.4458	0.0022	0.0089
ConcomitantHCQ	0.2420	0.2314	0.2399	0.4273	0.0021	0.0085
ConcomitantLFN	0.1801	0.1807	0.1781	0.3829	0.0019	0.0077

© 2023 Deakin CT et al. *JAMA Network Open.*

Characteristic	Weighted mean (ADA)	Weighted mean (TOF)	Unweighted mean (entire sample)	Unweighted standard deviation (entire sample)	Maximum permitted difference between means (ADA)	Maximum permitted difference between means (TOF)
ConcomitantSFZ	0.1502	0.1592	0.1520	0.3593	0.0018	0.0072
ConcomitantOCS	0.4930	0.4805	0.4905	0.5002	0.0025	0.0100
DAS28CRP_Time.0	5.2607	5.2413	5.2671	1.2885	0.0064	0.0258

© 2023 Deakin CT et al. *JAMA Network Open.*

eResults 4: Weighted Components of the DAS28CRP

The weighted means for each of the individual DAS28CRP components were summarised by treatment group on their original scales for each of the timepoints. It does not appear that any of the DAS28CRP components are driving the treatment effect.

Distributions of DAS28CRP components by treatment group at 0 months

eTable 18. Weighted means of DAS28CRP components at 0 months by treatment group for each imputed dataset

Imputation	SJC28 (ADA)	SJC28 (TOF)	TJC28 (ADA)	TJC28 (TOF)	CRP (ADA)	CRP (TOF)	Patient global (ADA)	Patient global (TOF)
1	13	13	14	14	12	12	57	55
2	13	13	14	14	12	12	58	56
3	13	13	14	14	11	13	57	56
4	13	13	14	14	12	12	56	56
5	13	13	14	14	12	12	57	56
6	13	13	14	14	12	12	58	55
7	13	13	14	14	11	11	57	54
8	13	13	14	14	11	12	56	55
9	13	13	14	14	12	12	57	56
10	13	13	13	14	12	12	57	52

Distributions of DAS28CRP components by treatment group at 3 months

eTable 19. Weighted means of DAS28CRP components at 3 months by treatment group for each imputed dataset

Imputation	SJC28 (ADA)	SJC28 (TOF)	TJC28 (ADA)	TJC28 (TOF)	CRP (ADA)	CRP (TOF)	Patient global (ADA)	Patient global (TOF)
1	2	1	2	1	7	6	28	26

© 2023 Deakin CT et al. *JAMA Network Open.*

Imputation	SJC28 (ADA)	SJC28 (TOF)	TJC28 (ADA)	TJC28 (TOF)	CRP (ADA)	CRP (TOF)	Patient global (ADA)	Patient global (TOF)
2	2	1	2	1	7	7	28	24
3	2	1	2	1	7	6	27	25
4	1	1	2	1	7	6	29	26
5	2	1	2	1	7	6	27	24
6	2	1	2	1	7	6	29	27
7	1	1	2	2	7	6	28	25
8	2	1	2	1	7	6	27	24
9	1	1	2	1	7	6	28	27
10	2	1	2	1	7	6	28	23

Distributions of DAS28CRP components by treatment group at 9 months

eTable 20. Weighted means of DAS28CRP components at 9 months by treatment group for each imputed dataset

Imputation	SJC28 (ADA)	SJC28 (TOF)	TJC28 (ADA)	TJC28 (TOF)	CRP (ADA)	CRP (TOF)	Patient global (ADA)	Patient global (TOF)
1	1	1	1	1	6	6	24	24
2	1	1	2	1	6	7	24	22
3	1	1	2	1	5	6	23	23
4	1	1	1	1	5	6	24	22
5	1	1	1	1	5	6	23	22
6	1	1	2	1	5	6	25	24
7	1	1	1	1	5	6	25	23
8	1	1	2	1	5	6	25	22
9	1	1	1	1	6	6	25	22
10	1	1	2	1	5	6	23	23

eResults 5: Point Estimates

Weighted and unweighted DAS28CRP at time zero and follow-up

Within each imputed dataset, baseline DAS28CRP and DAS28CRP at 3 and at 9 months were multiplied by the weights for each individual. The weighted mean DAS28CRP in each treatment group at these timepoints are summarised in eTable 21, along with their respective unweighted values. These estimates calculated within each imputed dataset were then pooled by taking the mean.

eTable 21. Weighted and unweighted DAS28CRP at baseline and at 3 and 9 months follow-up for ADA and TOF treatment groups

	ADA (n=569)	TOF (n=273)
Unweighted DAS28CRP at 0 months	5.3	5.2
Weighted DAS28CRP at 0 months	5.3	5.3
Unweighted DAS28CRP at 3 months	2.6	2.5
Weighted DAS28CRP at 3 months	2.6	2.4
Unweighted DAS28CRP at 9 months	2.3	2.3
Weighted DAS28CRP at 9 months	2.3	2.3

Average treatment effect

The difference in mean weighted DAS28CRP for TOF versus mean weighted DAS28CRP for ADA was calculated at 3 and at 9 months within each imputed dataset, with estimates for the ATE pooled by taking the mean (see code).

© 2023 Deakin CT et al. *JAMA Network Open.*

eResults 6: E-Values to Quantify Sensitivity of Results to Possible Unmeasured Confounding

The E-value is the minimum strength of association, on the risk ratio scale, that an unmeasured confounder or set of confounders would need to have with both the treatment and outcome, conditional on the measured covariates, to fully explain away a specific treatment-outcome association [11]. This can also be thought of as quantifying how strong unmeasured confounding would have to be in order to negate the observed results.

Point estimates and E-values for the ATE at 3 and 9 months on the risk ratio scale are given in eTable 22.

eTable 22. Point estimates and E-values on the risk ratio scale

Follow-up timepoint	Point estimate	E-value
3 months	0.92	1.39
9 months	0.99	1.13

These E-values imply that a modest effect size for an unmeasured confounder could potentially explain away the reported ATEs.

eResults 7: Drug Cessations Due to Adverse Reactions

Cessations of ADA or TOF that were recorded as being due to an adverse reaction were described for all eligible patients for all recorded follow-up, as well as the patients who were excluded from the analysis of effectiveness because of missing components of the DAS28CRP (total n=1168).

There were a total of 47 cessations due to an adverse reaction, of which 9 were considered non-serious (and included hepatotoxicity, recurrent skin infections, rashes, an allergic reaction, pruritus, nausea, tinnitus and nightmares). The remaining adverse reactions are described in eTable 10 in order of seriousness. Data on whether the adverse reactions were related to treatment were incomplete.

An adverse reaction recorded using the International Classification of Diseases 10 (ICD10) codes C* or D00-D48 was considered to be a malignancy [12]. A major cardiovascular event (MACE) was defined according to previously published the ICD10 codes for MACE in the context of administrative datasets and rheumatology: I21-I24 (myocardial infarction), I63-I66 (stroke), I11, I50, I97.1 (heart failure), Z95 (coronary artery bypass grafting) [13] [14].

© 2023 Deakin CT et al. *JAMA Network Open.*

eTable 23. Adverse reactions leading to cessations of ADA or TOF

Description of adverse reaction	ICD10	Severity of adverse reaction	Treatment group	Relation to treatment	Duration of treatment (months)
Embolic stroke	I63.4	Medically Significant	Tofacitinib	Probable	14.6
Pulmonary embolism	I26.9	Life-Threatening	Adalimumab	Unrelated	3.2
Pulmonary embolism	I26.9	Medically Significant	Tofacitinib	Probable	3.5
Deep vein thrombosis, lower limb/s	I80.2	Medically Significant	Tofacitinib		6.5
H. Zoster (shingles)	B02.8	Initial / Prolonged Hospitalisation	Adalimumab		13.6
Bacterial infection	A49.9	Medically Significant	Tofacitinib	Definite	3.2
Community acquired pneumonia	J18.9	Medically Significant	Tofacitinib		20.0
Pneumocystis jiroveci (carinii) pneumonia	B59	Medically Significant	Tofacitinib		6.2
Pneumonia	J18.9	Medically Significant	Adalimumab	Possible	44.7
Reaction to transfusion / infusion / therapeutic injection	T80.9	Medically Significant	Adalimumab		5.2
Reaction to transfusion / infusion / therapeutic injection	T80.9	Medically Significant	Adalimumab		5.3

© 2023 Deakin CT et al. *JAMA Network Open.*

Description of adverse reaction	ICD10	Severity of adverse reaction	Treatment group	Relation to treatment	Duration of treatment (months)
Reaction to transfusion / infusion / therapeutic injection	T80.9	Medically Significant	Adalimumab		8.1
Reaction to transfusion / infusion / therapeutic injection	T80.9	Medically Significant	Adalimumab	Probable	3.4
Reaction to transfusion / infusion / therapeutic injection	T80.9	Medically Significant	Adalimumab	Probable	20.1
Local skin eruption due to drug	L27.1	Medically Significant	Adalimumab	Probable	8.2
Local skin eruption due to drug	L27.1	Medically Significant	Adalimumab	Probable	5.0
Generalised skin eruption due to drug	L27.0	Persistent or Significant Disability	Adalimumab		23.2
Rash	R21	Medically Significant	Adalimumab	Probable	14.7
Rash	R21	Medically Significant	Adalimumab	Probable	10.4
Interstitial lung disease	J84.9	Persistent or Significant Disability	Adalimumab		6.9
Sarcoidosis	D86.9	Medically Significant	Adalimumab		22.8
Encephalitis, myelitis and encephalomyelitis in other diseases classified elsewhere	G05.8	Initial / Prolonged Hospitalisation	Adalimumab	Definite	13.9

© 2023 Deakin CT et al. *JAMA Network Open.*

Description of adverse reaction	ICD10	Severity of adverse reaction	Treatment group	Relation to treatment	Duration of treatment (months)
Guttate psoriasis	L40.4	Medically Significant	Adalimumab	Definite	4.1
Folliculitis	L73.9	Medically Significant	Tofacitinib		3.2
Headache	R51	Medically Significant	Tofacitinib		6.5
Abdominal pain	R10.4	Medically Significant	Adalimumab		14.6
Neuropathic pain	M79.29	Medically Significant	Adalimumab		9.0
Neuropathic pain	M79.29	Medically Significant	Tofacitinib		4.4
Peripheral neuropathy	G62.9	Medically Significant	Adalimumab	Possible	2.0
Drug-induced tremor	G25.1	Medically Significant	Tofacitinib	Definite	3.0
Organic psychosis	F09	Medically Significant	Tofacitinib	Possible	7.9
Nausea and vomiting	R11	Medically Significant	Tofacitinib		3.7
Nausea and vomiting	R11	Medically Significant	Tofacitinib	Probable	3.3

© 2023 Deakin CT et al. *JAMA Network Open.*

Description of adverse reaction	ICD10	Severity of adverse reaction	Treatment group	Relation to treatment	Duration of treatment (months)
Abnormal liver function tests	R79.8	Medically Significant	Tofacitinib	Probable	11.0
Hepatotoxicity	K71.9	Medically Significant	Tofacitinib		7.8
Drug-induced neutropenia	D70	Medically Significant	Adalimumab	Possible	63.3
Drug-induced thrombocytopenia	D69.5	Medically Significant	Tofacitinib	Probable	9.2
Mouth ulcers	K12.1	Medically Significant	Tofacitinib		3.5

© 2023 Deakin CT et al. *JAMA Network Open.*

eAppendix: Code

Random forest multiple imputation

```
# data.preimpute is a dataframe of the baseline and follow-up data before imp
utation
# T is a treatment indicator: 1=TOF, 0=ADA
# baseline.covars is a character variable listing all baseline covariates in
Table S1 except the components DAS28CRP at baseline
# disease.activity.t0 is a character variable listing disease activity variab
les including the transformed DAS28CRP components at baseline: sqrt.SJC28_Tim
e.0, sqrt.TJC28_Time.0, log.CRP_Time.0, PatientGlobal_Time.0, PhysicianGlobal
_Time.0, ESR_Time.0
# similarly disease.activity.t3 and disease.activity.t9 are character variabl
es listing disease activity variables at 3 months and 9 months

## initial imputation using the default PMM method to generate a method objec
t

impute.pmm <- mice(data.preimpute[, c("T", baseline.covars, disease.activity.
t0, disease.activity.t3, disease.activity.t9)],
            m=1,
            seed=749,
            maxit=1
            )

# extract the method object
meth.rf <- impute.pmm$method

# edit the method object to specify random forest will be used for the diseas
e activity variables
meth.rf["sqrt.SJC28_Time.0"] <- "rf"
meth.rf["sqrt.SJC28_Time.3"] <- "rf"
meth.rf["sqrt.SJC28_Time.9"] <- "rf"
meth.rf["sqrt.TJC28_Time.0"] <- "rf"
meth.rf["sqrt.TJC28_Time.3"] <- "rf"
meth.rf["sqrt.TJC28_Time.9"] <- "rf"
meth.rf["PatientGlobal_Time.0"] <- "rf"
meth.rf["PatientGlobal_Time.3"] <- "rf"
meth.rf["PatientGlobal_Time.9"] <- "rf"
meth.rf["PhysicianGlobal_Time.0"] <- "rf"
meth.rf["PhysicianGlobal_Time.3"] <- "rf"
meth.rf["PhysicianGlobal_Time.9"] <- "rf"
meth.rf["log.CRP_Time.0"] <- "rf"
meth.rf["log.CRP_Time.3"] <- "rf"
```

© 2023 Deakin CT et al. *JAMA Network Open.*

```
meth.rf["log.CRP_Time.9"] <- "rf"
meth.rf["ESR_Time.0"] <- "rf"
meth.rf["ESR_Time.3"] <- "rf"
meth.rf["ESR_Time.9"] <- "rf"


## RF-MI with m=10

m <- 10

impute.rf <- mice(data.preimpute[, c("T", baseline.covars, disease.activity.t
0, disease.activity.t3, disease.activity.t9)],
            m=m,
            seed=749,
            maxit=5,
            method=meth.rf
            )


# extract the complete datasets in long form
data.postimpute.long <- complete(impute.rf, action="long")

# calculate DAS28CRP at times 0, 3, 9
data.postimpute.long$DAS28CRP_Time.0 <- (0.56*data.postimpute.long$sqrt.TJC28
_Time.0) + (0.28*data.postimpute.long$sqrt.SJC28_Time.0) + (0.36*data.postimp
ute.long$log.CRP_Time.0) + (0.014*data.postimpute.long$PatientGlobal_Time.0)
+ 0.96

data.postimpute.long$DAS28CRP_Time.3 <- (0.56*data.postimpute.long$sqrt.TJC28
_Time.3) + (0.28*data.postimpute.long$sqrt.SJC28_Time.3) + (0.36*data.postimp
ute.long$log.CRP_Time.3) + (0.014*data.postimpute.long$PatientGlobal_Time.3)
+ 0.96

data.postimpute.long$DAS28CRP_Time.9 <- (0.56*data.postimpute.long$sqrt.TJC28
_Time.9) + (0.28*data.postimpute.long$sqrt.SJC28_Time.9) + (0.36*data.postimp
ute.long$log.CRP_Time.9) + (0.014*data.postimpute.long$PatientGlobal_Time.9)
+ 0.96

# bring in PatientID variable and S, an indicator for whether patients have D
AS28CRP components at follow-up; coerce to numeric
data.postimpute.long <- cbind.data.frame(sapply(data.preimpute[, c("PatientID
", "S")], rep.int, times=10),
                                         data.postimpute.long
                                         )
data.postimpute.long$S <- as.numeric(data.postimpute.long$S)

# indicator to represent patients who receive TOF and whose outcomes were obs
erved at follow-up
data.postimpute.long$trt1 <- data.postimpute.long$T * data.postimpute.long$S
```

© 2023 Deakin CT et al. *JAMA Network Open.*

```
# equals 1 if (T,S)=(1,1)

# indicator to represent patients who receive ADA and whose outcomes were obs
erved at follow-up
data.postimpute.long$trt0 <- (1 - data.postimpute.long$trt1) * data.postimput
e.long$S # equals 1 if (T,S)=(0,1)

# indicator to represent patients who do NOT those who received TOF and whose
outcomes were observed at follow-up
data.postimpute.long$missind1 <- 1 - data.postimpute.long$trt1

# indicator to represent patients who do NOT those who received ADA and whose
outcomes were observed at follow-up
data.postimpute.long$missind0 <- 1 - data.postimpute.long$trt0

# split imputed data into a list of imputed datasets
data.postimpute <- split(data.postimpute.long, f=data.postimpute.long$.imp)
```

Stable balancing weights

```
# list of balancing requirements for sbw
bal <- list()

bal$bal_cov <- c(baseline.covars, "DAS28CRP_Time.0") # which variables need t
o be balanced
bal$bal_std <- "target" # what is the `target' sample - setting it equal to "
target" means that the `target' sample is the entire sample
bal$bal_alg <- TRUE # tells sbw to use the algorithm
bal$bal_tol <- NULL
bal$bal_gri = c(0.002, 0.005,0.01,0.02,0.05,0.1,0.25,0.4) #grid of values to
use for tolerance
bal$bal_sam = 1000 # keep the default resampling procedure


# run sbw for patients who receive ADA and whose outcomes are observed
sbw.choose0 <- vector(mode="list", length=m)

for(j in 1:m) {

  sbw.choose0[[j]] <- sbw(dat=data.postimpute[[j]], ind="missind0", out=NULL,
bal = bal, sol = list(sol_nam = "quadprog"), par = list(par_est = "pop"))

  data.postimpute[[j]]$sbw.wei0 <- sbw.choose0[[j]]$dat_weights$sbw_weights #
get the weights

}
```

© 2023 Deakin CT et al. *JAMA Network Open.*

```
# run sbw for patients who receive TOF and whose outcomes are observed
sbw.choose1 <- vector(mode="list", length=m)

for(j in 1:m) {

  sbw.choose1[[j]] <- sbw(dat=data.postimpute[[j]], ind="missind1", out=NULL,
bal = bal, sol = list(sol_nam = "quadprog"), par = list(par_est = "pop"))

  data.postimpute[[j]]$sbw.wei1 <- sbw.choose1[[j]]$dat_weights$sbw_weights #
get the weights

}
```

Point estimates

```
ate.3m <- vector(mode="list", length=m)

for(j in 1:m) {
  ate.3m[[j]] <- xy.weighed.mean1[[j]]["DAS28CRP_Time.3"] - xy.weighed.mean0[
[j]]["DAS28CRP_Time.3"]
}

mean(unlist(ate.3m))


ate.9m <- vector(mode="list", length=m)

for(j in 1:m) {
  ate.9m[[j]] <- xy.weighed.mean1[[j]]["DAS28CRP_Time.9"] - xy.weighed.mean0[
[j]]["DAS28CRP_Time.9"]
}

mean(unlist(ate.9m))
```

Percentile method for 95% confidence interval

```
#xy.weighed.mean1 is a list of lists of weighted means for patients on TOF
#xy.weighed.mean0 is a list of lists of weighted means for patients on TOF

alpha <- 0.05 #alpha to use for statistical significance
null.pvalue <- 0 #null value for ATE

## 3 months

ate.3m <- vector(mode="list", length=B) #list to store list of ATEs for each
```

© 2023 Deakin CT et al. *JAMA Network Open.*

```
imputed dataset for each bootstrap sample
ate.3m.boot <- vector(mode="list", length=B) #list to store ATEs for each boo
tstrap sample

for(i in 1:B) {
  ate.3m[[i]] <- vector(mode="list", length=B)
  for(j in 1:m) {
    ate.3m[[i]][[j]] <- xy.weighed.mean1[[i]][[j]]["DAS28CRP_Time.3"] - xy.we
ighed.mean0[[i]][[j]]["DAS28CRP_Time.3"]
  }
  ate.3m.boot[[i]] <- mean(unlist(ate.3m[[i]]))
}

ate.3m.boot <- unlist(ate.3m.boot)

ate.3.ci <- quantile(ate.3m.boot, probs =c(alpha/2, 1-alpha/2)) #lower and up
per limits for 95% CI

ate.3.halfpval <- mean(ate.3m.boot > null.pvalue + 0.5*mean(ate.3m.boot == nu
ll.pvalue)) #half p-value
ate.3.pval <- 2*min(c(ate.3.halfpval,1-ate.3.halfpval)) #p-value

## 9 months

ate.9m <- vector(mode="list", length=B) #list to store list of ATEs for each
imputed dataset for each bootstrap sample
ate.9m.boot <- vector(mode="list", length=B) #list to store ATEs for each boo
tstrap sample

for(i in 1:B) {
  ate.9m[[i]] <- vector(mode="list", length=B)
  for(j in 1:m) {
    ate.9m[[i]][[j]] <- xy.weighed.mean1[[i]][[j]]["DAS28CRP_Time.9"] - xy.we
ighed.mean0[[i]][[j]]["DAS28CRP_Time.9"]
  }
  ate.9m.boot[[i]] <- mean(unlist(ate.9m[[i]]))
}

ate.9m.boot <- unlist(ate.9m.boot)

ate.9.ci <- quantile(ate.9m.boot, probs =c(alpha/2, 1-alpha/2)) #lower and up
per limits for 95% CI

ate.9.halfpval <- mean(ate.9m.boot > null.pvalue + 0.5*mean(ate.9m.boot == nu
ll.pvalue)) #half p-value
ate.9.pval <- 2*min(c(ate.9.halfpval,1-ate.9.halfpval)) #p-value
```

© 2023 Deakin CT et al. *JAMA Network Open.*

Session Information

```
## R version 4.0.2 (2020-06-22)
## Platform: x86_64-apple-darwin17.0 (64-bit)
## Running under: macOS  10.16
##
## Matrix products: default
## BLAS:   /Library/Frameworks/R.framework/Versions/4.0/Resources/lib/libRbla
s.dylib
## LAPACK: /Library/Frameworks/R.framework/Versions/4.0/Resources/lib/libRlap
ack.dylib
##
## locale:
## [1] en_AU.UTF-8/en_AU.UTF-8/en_AU.UTF-8/C/en_AU.UTF-8/en_AU.UTF-8
##
## attached base packages:
## [1] stats     graphics  grDevices utils     datasets  methods   base
##
## other attached packages:
##  [1] survival_3.2-11 gridExtra_2.3   EValue_4.1.3    mice_3.14.0
##  [5] cobalt_4.3.1    ggdag_0.2.5     dagitty_0.3-1   flextable_0.6.6
##  [9] forcats_0.5.1   stringr_1.4.0   dplyr_1.0.7     purrr_0.3.4
## [13] readr_1.4.0     tidyr_1.1.3     tibble_3.1.4    ggplot2_3.3.6
## [17] tidyverse_1.3.1
##
## loaded via a namespace (and not attached):
##  [1] nlme_3.1-152       fs_1.5.0           MetaUtility_2.1.2  lubridate_1.
7.10
##  [5] httr_1.4.2         tools_4.0.2        backports_1.4.1    utf8_1.2.2
##  [9] R6_2.5.1           metafor_3.0-2      DBI_1.1.1          colorspace_2
.0-1
## [13] withr_2.4.2        tidyselect_1.1.1   curl_4.3.2         compiler_4.0
.2
## [17] cli_3.0.1          rvest_1.0.0        xml2_1.3.2         officer_0.3.
18
## [21] labeling_0.4.2     scales_1.1.1       systemfonts_1.0.2  digest_0.6.2
7
## [25] rmarkdown_2.18     base64enc_0.1-3    pkgconfig_2.0.3    htmltools_0.
5.3
## [29] highr_0.9          dbplyr_2.1.1       fastmap_1.1.0      rlang_0.4.11
## [33] readxl_1.3.1       rstudioapi_0.13    farver_2.1.0       generics_0.1
.0
## [37] jsonlite_1.7.2     zip_2.1.1          magrittr_2.0.1     Matrix_1.3-3
## [41] Rcpp_1.0.7         munsell_0.5.0      fansi_0.5.0        viridis_0.6.
1
## [45] gdtools_0.2.3      lifecycle_1.0.0    stringi_1.7.4      yaml_2.2.1
## [49] ggraph_2.0.6       mathjaxr_1.4-0     MASS_7.3-54        grid_4.0.2
## [53] ggrepel_0.9.1      crayon_1.4.1       lattice_0.20-44    graphlayouts
_0.8.0
```

© 2023 Deakin CT et al. *JAMA Network Open.*

```
## [57] haven_2.4.1        splines_4.0.2      hms_1.1.0          knitr_1.33
## [61] pillar_1.6.2       igraph_1.2.6       uuid_0.1-4         boot_1.3-28
## [65] reprex_2.0.1       glue_1.4.2         evaluate_0.14      V8_3.4.2
## [69] data.table_1.14.0  modelr_0.1.8       tweenr_1.0.2       vctrs_0.3.8
## [73] cellranger_1.1.0   polyclip_1.10-0    gtable_0.3.0       assertthat_0
.2.1
## [77] ggforce_0.3.3      xfun_0.34          broom_0.7.9        tidygraph_1.
2.1
## [81] viridisLite_0.4.0  ellipsis_0.3.2
```

© 2023 Deakin CT et al. *JAMA Network Open.*

References

1 Hernán MA, Robins JM. Using Big Data to Emulate a Target Trial When a Randomized Trial Is Not Available. *American Journal of Epidemiology* 2016;**183**:758–64. doi:10.1093/aje/kwv254

2 Littlejohn GO, Tymms KE, Smith T *et al.* Using big data from real-world Australian rheumatology encounters to enhance clinical care and research. *Clinical and Experimental Rheumatology* 2020;**38**:874–80.https://www.clinexprheumatol.org/abstract.asp?a=14467

3 Pearl J. Causal diagrams for empirical research. *Biometrika* 1995;**82**:669–88. doi:10.1093/biomet/82.4.669

4 Textor J, Zander B van der, Gilthorpe MS *et al.* Robust causal inference using directed acyclic graphs: the R package 'dagitty'. *International Journal of Epidemiology* 2016;**45**:1887–94. doi:10.1093/ije/dyw341

5 Barrett M. ggdag: Analyze and Create Elegant Directed Acyclic Graphs. 2022.

6 Buuren S van, Groothuis-Oudshoorn K. mice: Multivariate Imputation by Chained Equations in R. *Journal of Statistical Software* 2011;**45**:1–67. doi:10.18637/jss.v045.i03

7 Zubizarreta JR. Stable Weights that Balance Covariates for Estimation With Incomplete Outcome Data. *Journal of the American Statistical Association* 2015;**110**:910–22. doi:10.1080/01621459.2015.1023805

8 Chattopadhyay A, Hase CH, Zubizarreta JR. Balancing vs modeling approaches to weighting in practice. *Statistics in Medicine* 2020;**39**:3227–54. doi:https://doi.org/10.1002/sim.8659

9 Bartlett JW, Hughes RA. Bootstrap inference for multiple imputation under uncongeniality and misspecification. *Statistical Methods in Medical Research* 2020;**29**:3533–46. doi:10.1177/0962280220932189

10 Zubizarreta JR, Li Y, Kim K. sbw: Stable Balancing Weights for Causal Inference and Missing Data. 2021.

11 VanderWeele TJ, Ding P. Sensitivity Analysis in Observational Research: Introducing the E-Value. *Annals of Internal Medicine* 2017;**167**:268–74. doi:10.7326/M16-2607

12 Organization WH. *ICD-10 : international statistical classification of diseases and related health problems / World Health Organization*. Geneva:: World Health Organization 2004.

13 Thurah A de, Andersen IT, Tinggaard AB *et al.* Risk of major adverse cardiovascular events among patients with rheumatoid arthritis after initial CT-based diagnosis and treatment. *RMD Open* 2020;**6**:e001113. doi:10.1136/rmdopen-2019-001113

© 2023 Deakin CT et al. *JAMA Network Open.*

14 Bosco E, Hsueh L, McConeghy KW *et al.* Major adverse cardiovascular event definitions used in observational analysis of administrative databases: a systematic review. *BMC Medical Research Methodology* 2021;**21**:241. doi:10.1186/s12874-021-01440-5

© 2023 Deakin CT et al. *JAMA Network Open.*
