# Supplementary material for: Comparative Effectiveness of Adalimumab vs Tofacitinib in Patients With Rheumatoid Arthritis in Australia
Source: JAMA Netw Open. 2023 Jun 29;6(6):e2320851. doi: 10.1001/jamanetworkopen.2023.20851 (PMC10311390; doi:10.1001/jamanetworkopen.2023.20851)
Supplement: Supplement 2. — OPAL Rheumatology Network [file jamanetwopen-e2320851-s002.pdf]

Supplemental Online Content: Nonauthor Collaborators
*First name, last name, and suffix (if applicable) are required and will appear in PubMed.

***Group Name(s):** OPAL Rheumatology Network							
***First Name and Middle Initial(s)**	***Last Name**	***Suffix (eg, Jr, III)**	Academic Degrees	Institution	Location (city, state/province, country)	Role or Contribution, eg, chair, principal investigator	Group (if more than 1 Group listed in the byline) and/or Subgroup (eg, Steering Committee)
Adam	Rischin		MBBS, FRACP	Malvern Rheumatology	VIC	Data collection	
Adam	Scott-Charlton		MBBS, FRACP	Rheumatology Tasmania	TAS	Data collection	
Alannah	Quinlivan		MBBS, FRACP	Southern Rheumatology	VIC	Data collection	
Alex	Stockman		MBBS, FRACP	Footscray Rheumatology Clinic	VIC	Data collection	
Alexandra	Capon		MBBS, FRACP	Orthopaedic & Arthritis Specialist Centre	NSW	Data collection	
Ana	Ananda		MBBS, FRACP	Hills Rheumatology	NSW	Data collection	
Andrew	Foote		MBBS, FRACP	Epping Specialist Group	VIC	Data collection	
Anna	Dorai-Raj		MBBS, FRACP	Rheumatology ACT	ACT	Data collection	
Anna	Finniss		MBBS, FRACP	Orthopaedic & Arthritis Specialist Centre	NSW	Data collection	
Aoife	Sweeney		MBBS, FRACP	Pacific Rheumatology	QLD	Data collection	
Armi	Salonga		MBBS, FRACP	Pacific Rheumatology	QLD	Data collection	
Arvin	Damodaran		MBBS, FRACP	Rheumatology Specialist Care	NSW	Data collection	
Ashleigh	Hennessey		MBBS, FRACP	Redcliffe and Northside Rheumatology	QLD	Data collection	
Bain	Shenstone		MBBS, FRACP	Hills Rheumatology	NSW	Data collection	

Supplemental Online Content: Nonauthor Collaborators
*First name, last name, and suffix (if applicable) are required and will appear in PubMed.

***First Name and Middle Initial(s)**	***Last Name**	***Suffix (eg, Jr, III)**	Academic Degrees	Institution	Location (city, state/province, country)	Role or Contribution, eg, chair, principal investigator	Group (if more than 1 Group listed in the byline) and/or Subgroup (eg, Steering Committee)
Barry	Kane		MBBS, FRACP	Hills Rheumatology	NSW	Data collection	
Benjamin	Sutu		MBBS, FRACP	Royal Melbourne Hospital	VIC	Data collection	
Bita	Omidvar		MBBS, FRACP	Southern Rheumatology	VIC	Data collection	
Champa	Nataraja		MBBS, FRACP	Peninsual Rheumatology	VIC	Data collection	
Charles	Inderjeeth		MBBS, FRACP	Subiaco Rheumatology	WA	Data collection	
Chiwai	Tong		MBBS, FRACP	Orthopaedic & Arthritis Specialist Centre	NSW	Data collection	
Chris	Mack		MBBS, FRACP	Coast Joint Care & Lilydale Private Hospital	QLD	Data collection	
Claire	Barrett		MBBS, FRACP	Redcliffe and Northside Rheumatology	QLD	Data collection	
Dan	Sumpton		MBBS, FRACP	Hills Rheumatology	NSW	Data collection	
Daniel	Boulos		MBBS, FRACP	Southern Rheumatology	VIC	Data collection	
Daniel	Lewis		MBBS, FRACP	Rheumatology Centre	VIC	Data collection	
Dave	Nicholls		MBBS, FRACP	Coast Joint Care	QLD	Data collection	
David	Mathers		MBBS, FRACP	Georgetown Arthritis	NSW	Data collection	
Deb	Speden		MBBS, FRACP	Rheumatology Tasmania	TAS	Data collection	
Diana	Chessman		MBBS, FRACP	Orthopaedic & Arthritis Specialist Centre	NSW	Data collection	

Supplemental Online Content: Nonauthor Collaborators
*First name, last name, and suffix (if applicable) are required and will appear in PubMed.

***First Name and Middle Initial(s)**	***Last Name**	***Suffix (eg, Jr, III)**	Academic Degrees	Institution	Location (city, state/province, country)	Role or Contribution, eg, chair, principal investigator	Group (if more than 1 Group listed in the byline) and/or Subgroup (eg, Steering Committee)
Dickson	MA		MBBS, FRACP	Barwon Rheumatology	VIC	Data collection	
Emily	Ong		MBBS, FRACP	Peninsula Rheumatology	VIC	Data collection	
Evange	Romas		MBBS, FRACP	Melbourne Arthritis Associates	VIC	Data collection	
Frank	Laska		MBBS, FRACP	Footscray Rheumatology Clinic	VIC	Data collection	
Fred	Joshua		MBBS, FRACP	Rheumatology Specialist Care	NSW	Data collection	
Gemma	Strickland		MBBS, FRACP	Barwon Rheumatology	VIC	Data collection	
Gene-Siew	Ngian		MBBS, FRACP	Northern Rheumatology	VIC	Data collection	
Geoff	Littlejohn		MBBS, FRACP	Monash University	VIC	Data collection	
Gerald	Tracey		MBBS, FRACP	Pacific Rheumatology	QLD	Data collection	
Hedley	Griffiths		MBBS, FRACP	Barwon Rheumatology	VIC	Data collection	
Helen	Cooley		MBBS, FRACP	Hobart Specialist Group	TAS	Data collection	
Ildiko	Telegdy		MBBS, FRACP	Townsville Hospital	QLD	Data collection	
Ingrid	Hutton		MBBS, FRACP	Coast Joint Care	QLD	Data collection	
Jacky	Chay		MBBS, FRACP	Pacific Rheumatology	QLD	Data collection	
Jane	Oliver		MBBS, FRACP	Hornsby	NSW	Data collection	

*First name, last name, and suffix (if applicable) are required and will appear in PubMed.

***First Name and Middle Initial(s)**	***Last Name**	***Suffix (eg, Jr, III)**	Academic Degrees	Institution	Location (city, state/province, country)	Role or Contribution, eg, chair, principal investigator	Group (if more than 1 Group listed in the byline) and/or Subgroup (eg, Steering Committee)
Jane	Zochling		MBBS, FRACP	Rheumatology Tasmania	TAS	Data collection	
Jason	Ly		MBBS, FRACP	Townsville Hospital	QLD	Data collection	
Jayne	Moxey		MBBS, FRACP	Royal Melbourne Hospital	VIC	Data collection	
Jennifer	Harmer		MBBS, FRACP	Cabrini Malvern	VIC	Data collection	
Jeremy	Wang		MBBS, FRACP	Susan Street Specialists Centre	NSW	Data collection	
Jianna	He		MBBS, FRACP	Shire Specialists	NSW	Data collection	
Joanna	Hall		MBBS, FRACP	Barwon Rheumatology	VIC	Data collection	
John	May		MBBS, FRACP	Barwon Rheumatology	VIC	Data collection	
John	Moi		MBBS, FRACP	Royal Melbourne Private	VIC	Data collection	
John	vander-Kallen		MBBS, FRACP	Georgetown Arthritis	NSW	Data collection	
Juan	Aw		MBBS, FRACP	Peninsual Rheumatology	VIC	Data collection	
Karen	Pui		MBBS, FRACP	Townsville Hospital	QLD	Data collection	
Kate	Franklyn		MBBS, FRACP	Malvern Rheumatology	VIC	Data collection	
Kate	Gregory-Wong		MBBS, FRACP	Shire specialists	NSW	Data collection	
Kathy	Tymms		MBBS, FRACP	Canberra Rheumatology	ACT	Data collection	

Supplemental Online Content: Nonauthor Collaborators
*First name, last name, and suffix (if applicable) are required and will appear in PubMed.

***First Name and Middle Initial(s)**	***Last Name**	***Suffix (eg, Jr, III)**	Academic Degrees	Institution	Location (city, state/province, country)	Role or Contribution, eg, chair, principal investigator	Group (if more than 1 Group listed in the byline) and/or Subgroup (eg, Steering Committee)
Katie	Morrisroe		MBBS, FRACP	Rheumatology ACT	ACT	Data collection	
Katy	Over		MBBS, FRACP	Wangratta	VIC	Data collection	
Ken	Cai		MBBS, FRACP	Shire Specialists	NSW	Data collection	
Ken	Khoo		MBBS, FRACP	Rheumatology ACT	ACT	Data collection	
Ken	Maguire		MBBS, FRACP	Selangor Medical Centre	QLD	Data collection	
Kiri	Langford		MBBS, FRACP	Orthopaedic & Arthritis Specialist Centre	NSW	Data collection	
Kokum	Dissanayake		MBBS, FRACP	Canberra Rheumatology	ACT	Data collection	
Kristy	Yap		MBBS, FRACP	Peninsula Rheumatology	VIC	Data collection	
Laila	Girgis		MBBS, FRACP	Heart Lung Rheumatology	NSW	Data collection	
Laurel	Young		MBBS, FRACP	Redcliffe and Northside Rheumatology	QLD	Data collection	
Leanne	Alblas		MBBS, FRACP	Northern Rheumatology	VIC	Data collection	
Les	Barnsley		MBBS, FRACP	Concord and West Gosford	NSW	Data collection	
Leticia	Deveza		MBBS, FRACP	Rheumatology Specialist Care	NSW	Data collection	
Louisa	Voight		MBBS, FRACP	Coast Joint Care	QLD	Data collection	
Lucy	Croyle		MBBS, FRACP	Barwon Rheumatology	VIC	Data collection	

***First Name and Middle Initial(s)**	***Last Name**	***Suffix (eg, Jr, III)**	Academic Degrees	Institution	Location (city, state/province, country)	Role or Contribution, eg, chair, principal investigator	Group (if more than 1 Group listed in the byline) and/or Subgroup (eg, Steering Committee)
Malcolm	Handel		MBBS, FRACP	St Vincents Hospital	NSW	Data collection	
Malcolm	Turner		MBBS, FRACP	Hobart Specialist Group	TAS	Data collection	
Maninder	Mundae		MBBS, FRACP	Northern Rheumatology	VIC	Data collection	
Maree	Micallef		MBBS, FRACP	Royal Melbourne Hospital	VIC	Data collection	
Marie	Feletar		MBBS, FRACP	Dandenong	VIC	Data collection	
Mark	Arnold		MBBS, FRACP	Dubbo/Orange	NSW	Data collection	
Mark	Collins		MBBS, FRACP	Boradmeadow	NSW	Data collection	
Mathew	Reynolds		MBBS, FRACP	Hobart Specialist Group	TAS	Data collection	
Maxine	Isbel		MBBS, FRACP	New WA Practice	WA	Data collection	
Maxine	Szramka		MBBS, FRACP	Footscray	NSW	Data collection	
Melinda	Wong		MBBS, FRACP	Cabrini	VIC	Data collection	
Mona	Marabani		MBBS, FRACP	Campsie	NSW	Data collection	
Mueed	Mian		MBBS, FRACP	Northern Rheumatology	VIC	Data collection	
Mueed	Mian		MBBS, FRACP	Epping Specialist Group	VIC	Data collection	
Nigel	Wood		MBBS, FRACP	Barwon Rheumatology	VIC	Data collection	

Supplemental Online Content: Nonauthor Collaborators
*First name, last name, and suffix (if applicable) are required and will appear in PubMed.

***First Name and Middle Initial(s)**	***Last Name**	***Suffix (eg, Jr, III)**	Academic Degrees	Institution	Location (city, state/province, country)	Role or Contribution, eg, chair, principal investigator	Group (if more than 1 Group listed in the byline) and/or Subgroup (eg, Steering Committee)
Pallavi	Shamdasani		MBBS, FRACP	Epping Specialist Group	VIC	Data collection	
Paul	Bird		MBBS, FRACP	Rheumatology Specialist Care	NSW	Data collection	
Paul	Mansfield		MBBS, FRACP	Georgetown Arthritis	NSW	Data collection	
Peta	Pentony		MBBS, FRACP	Rheumatology ACT	ACT	Data collection	
Pete	Nash		MBBS, FRACP	Coast Joint Care	QLD	Data collection	
Peter	Youssef		MBBS, FRACP	Susan Street Specialists Centre	NSW	Data collection	
Rachelle	Buchbinder		MBBS, FRACP	Malvern Rheumatology	VIC	Data collection	
Ross	Penglase		MBBS, FRACP	Heart Lung Rheumatology	NSW	Data collection	
Ruvinka	Jayalath		MBBS, FRACP	Townsville Hospital	QLD	Data collection	
Sabina	Ciciriello		MBBS, FRACP	Royal Melbourne Hospital	VIC	Data collection	
Sarina	Kempe		MBBS, FRACP	Coast Joint Care	QLD	Data collection	
Sayed	Fayez		MBBS, FRACP	Townsville Hospital	QLD	Data collection	
Shereen	Oon		MBBS, FRACP	Royal Melbourne Hospital	VIC	Data collection	
Shreeya	Patel		MBBS, FRACP	Northern Rheumatology	VIC	Data collection	
Shunil	Sharma		MBBS, FRACP	John Flynn Private	QLD	Data collection	

Supplemental Online Content: Nonauthor Collaborators
*First name, last name, and suffix (if applicable) are required and will appear in PubMed.

***First Name and Middle Initial(s)**	***Last Name**	***Suffix (eg, Jr, III)**	Academic Degrees	Institution	Location (city, state/province, country)	Role or Contribution, eg, chair, principal investigator	Group (if more than 1 Group listed in the byline) and/or Subgroup (eg, Steering Committee)
Simon	Chatfield		MBBS, FRACP	Royal Melbourne Hospital	VIC	Data collection	
Sonam	Joshi		MBBS, FRACP	Shunil Sharma's practice	QLD	Data collection	
Stanley	Seah		MBBS, FRACP	Rheumatology United Penrith,	NSW	Data collection	
Steve	Truong		MBBS, FRACP	Coast Joint Care	QLD	Data collection	
Suren	Jayaweera		MBBS, FRACP	Rheumatology ACT	ACT	Data collection	
Talib	Tahir		MBBS, FRACP	Coburg	VIC	Data collection	
Ted	Tsai		MBBS, FRACP	Rheumatology ACT	ACT	Data collection	
Tim	Godfrey		MBBS, FRACP	Cabrini Medical Centre	VIC	Data collection	
Tina	Racunica		MBBS, FRACP	Southern Rheumatology	VIC	Data collection	
Winston	Chang		MBBS, FRACP	Penninsula Rheumatology	VIC	Data collection	
Yuen	Loew		MBBS, FRACP	Southern Rheumatology	VIC	Data collection	
Michelle	Papandony		MBBS, FRACP	Cabrini Medical Centre	VIC	Data collection	
Yumi	Oh		MBBS, FRACP	Redcliff & Northside Rheumatology	QLD	Data collection	
