# Supplementary material for: Comparative Effectiveness of Adalimumab vs Tofacitinib in Patients With Rheumatoid Arthritis in Australia
Source: JAMA Netw Open. 2023 Jun 29;6(6):e2320851. doi: 10.1001/jamanetworkopen.2023.20851 (PMC10311390; doi:10.1001/jamanetworkopen.2023.20851)
Supplement: Supplement 3. — Data Sharing Statement [file jamanetwopen-e2320851-s003.pdf]

Data Sharing Statement

Deakin. Comparative Effectiveness of Adalimumab vs Tofacitinib in Patients With Rheumatoid Arthritis in Australia. *JAMA Netw Open*. Published June 29, 2023.
doi:10.1001/jamanetworkopen.2023.20851

Data
Data available: No

Additional Information
Explanation for why data not available: Full details of the study protocol and statistical analysis plan are provided in the Supplementary Material. Requests for access to summary statistics will be considered by the OPAL Scientific Review Committee.
